# Exploiting racemism enhanced organic room-temperature phosphorescence to demonstrate Wallach’s rule in the lighting chiral chromophores

**DOI:** 10.1038/s41467-020-15976-5

**Published:** 2020-05-01

**Authors:** Xiugang Wu, Chun-Ying Huang, Deng-Gao Chen, Denghui Liu, Chichi Wu, Keh-Jiunh Chou, Bin Zhang, Yafei Wang, Yu Liu, Elise Y. Li, Weiguo Zhu, Pi-Tai Chou

**Affiliations:** 1 School of Materials Science and Engineering, Jiangsu Collaborative Innovation Center of Photovoltaic Science and Engineering, Jiangsu Engineering Laboratory of Light-Electricity-Heat Energy-Converting Materials and Applications, Jiangsu Key Laboratories of Environment-Friendly Polymers, National Experimental Demonstration Center for Materials Science and Engineering, Changzhou University, Changzhou, China; 2 Department of Chemistry, National Taiwan University, No. 1, Sec. 4, Roosevelt Rd., Taipei, 10617 Taiwan, ROC; 3 Department of Chemistry, National Normal Taiwan University, No. 162, Sec. 1, Heping E. Rd., Taipei, 10610 Taiwan, ROC

**Keywords:** Circular dichroism, Electronic devices, Organic molecules in materials science

## Abstract

The correlation between molecular packing structure and its room-temperature phosphorescence (RTP), hence rational promotion of the intensity, remains unclear. We herein present racemism enhanced RTP chiral chromophores by 2,2-bis-(diphenylphosphino)-1,1-napthalene (rac-**BINAP**) in comparison to its chiral counterparts. The result shows that rac-**BINAP** in crystal with denser density, consistent with a long standing Wallach’s rule, exhibits deeper red RTP at 680 nm than that of the chiral counterparts. The cross packing between alternative R- and S- forms in rac-**BINAP** crystal significantly retards the bimolecular quenching pathway, triplet-triplet annihilation (TTA), and hence suppresses the non-radiative pathway, boosting the RTP intensity. The result extends the Wallach’s rule to the fundamental difference in chiral-photophysics. In electroluminescence, rac-**BINAP** exhibits more balanced fluorescence versus phosphorescence intensity by comparison with that of photoluminescence, rendering a white-light emission. The result paves an avenue en route for white-light organic light emitting diodes via full exploitation of intrinsic fluorescence and phosphorescence.

## Abstract (web summary)

Room temperature phosphorescence (RTP) is a topic which is currently widely studied, but a study on the correlation between types of molecular solid and RTP properties is lacking. Here the authors show that RTP is enhanced in the racemic form of axial chiral molecules due to its denser R- and S- cross packing arrangement.

## Introduction

Harvesting triplet excitons from an emitter in organic light-emitting diodes (OLEDs) has attracted considerable attentions from worldwide researchers, owing to its 75% proportion compared with singlet excitons upon electrical excitation^1–5^. Though many approaches, such as the transition metal complexes, thermally activated delayed fluorescence (TADF) molecules and triplet–triplet annihilation (TTA)^6^, have been applied to harvest the triplet excitons for luminescence, drawbacks for each technique still exist. For example, the transition metal complexes such as Ir (III) and Pt (II) may suffer from high cost, scarcity, and toxicity, hindering their industrial applications^7,8^. The emitters with TTA mechanism can convert two triplet excitons into one singlet exciton for fluorescence; however, the maximum internal quantum efficiency (IQE) is restricted to 62.5% in theory^9^.

Unlike TADF molecules, of which the energy gap between lowest lying singlet and triplet states (Δ*E*_S-T_) needs to be regulated^10^, organic molecules that are able to exhibit room-temperature phosphorescence (RTP) are not limited to the Δ*E*_S-T_ range that has to be thermally accessible^11–14^. Therefore, it is less demanding in the molecular design so long as one can reduce the non-radiative deactivation pathways in the triplet manifolds to boost RTP. Nevertheless, attempts to obtain an efficient non-metal organic RTP-based OLED device are still in the infant stage, especially with carbazole-free RTP emitters^5,15,16^. In yet another reserch direction, the long lifespan of RTP materials also shows potential applications in e.g., data encryption, optical recording devices, chemosensor, and bioimaging, etc.^11–14,17–19^.

Unfortunately, the long radiative lifetime also leads to more efficient quenching of the triplet exciton than that of the singlet exciton. In this aspect, an efficacious way to suppress the quench of long-lived triplet exciton is to inhibit the non-radiative pathways associated with those unimolecular large amplitude motions as well as the bimolecular quenching process such as TTA. This may be effectively operative by constructing a rigid environment such as host–guest system, polymer matrix, etc.^1,20–24^, so that the rate of triplet emission outcompetes the non-radiative deactivation. This strategy has triggered vast boom on the study of organic RTP materials in crystal^13^.

However, there is no report regarding the empirical correlation between types of molecular solid and RTP properties, such as intensity and/or relaxation dynamics at this stage. This could be a challenging task because it is expected to be of molecular structure dependence. Nonetheless, for the chiral molecules with similar structure and photophysical properties, it will be of fundamental importance to comprehend whether there is any correlation between RTP and crystal packing. This issue reminds us of an old, long-standing Wallach’s rule, stating that for the chiral molecule, the single crystal of a racemic form is always denser than their chiral counterparts^25–29^. In the case of chirality-racemism-dependent emission, for instance, Jin et al. reported the photophysical properties by mechanochromism between racemic and S-form of the gold (I) complex^30^.

Herein, we present the intrinsic difference for the chiral–photophysical property relationship, especially the non-metal-enhanced RTP, for which the emissive property is extremely sensitive to the packing density and the molecular arrangement due to its forbidden transition and hence exceedingly long lifetime. We propose the non-metal organic RTP, if there is any, to be enhanced in the racemic form of axial chiral molecules due to its denser R- and S-  forms  cross-packing arrangement. The results extend Wallach’s rule to lighting chiral chromophores in their intrinsic difference in chiral photophysics.

**Fig. 1:**
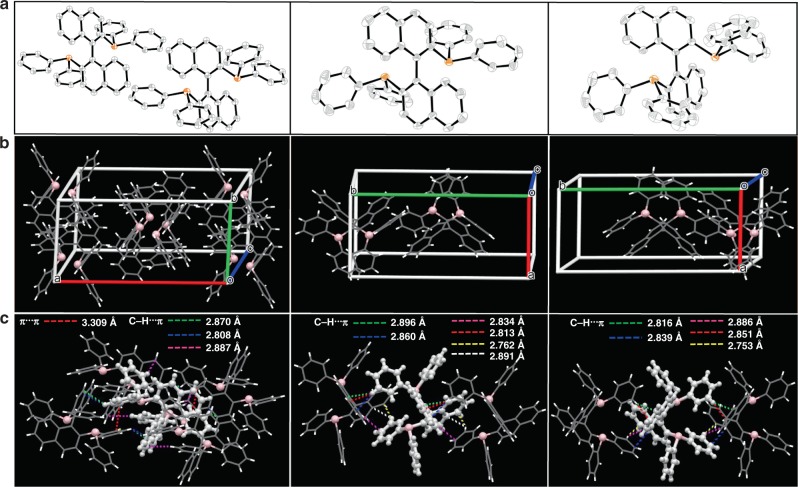
Structural information of BINAP in crystal. **a** Molecular structure of rac-**BINAP** (left), R- (central) and S-**BINAP** (right) in crystal. **b** Molecular packing of **BINAP** in different configurations from left (rac-**BINAP**), central (R-**BINAP**), and right (S-**BINAP**) in a crystal unit cell. **c** Intermolecular interactions of single molecule with adjacent ones from left (rac-**BINAP**), central (R-**BINAP**), and right (S-**BINAP**).

To verify the hypothesis, we have made great efforts in searching axial chiral molecules that exhibit RTP in the single crystal. The quest is mainly based on the chiral organic molecules anchoring non-metal heavy element that contributes to the emissive moiety. Accordingly, the phosphorus atom has received our attention because lone-pair electron and large atomic number (*Z* = 15) may be able to strengthen spin–orbit coupling (SOC) and facilitate intersystem crossing (ISC) between π→ π* and n→ π* character states (the El-Sayed rule)^31,32^. It turns out that the triphenyl phosphine-anchored compound, 2,2-bis-(diphenylphosphino)-1,1-napthalene (**BINAP**, Fig. 1), an important axial chirality molecule for asymmetric catalysis^33–35^, shows prominent deep-red RTP, which is previously unrecognized. In this study, the steady-state measurement clearly indicates that the racemic form of **BINAP** (rac-**BINAP**) shows nearly fourfold in the intensity of deep-red RTP compared with that of the respective chiral R (R-**BINAP)** and S (S-**BINAP**) forms. This observation correlates well with the denser packing mode, which results from the alternative and cross-R/S arrangement of rac-**BINAP** obeying Wallach’s rule, leading to the suppression of non-radiative pathways.

In addition, the family of **BINAP** possesses simultaneously bluish-green fluorescence and deep-red phosphorescence, showing the potential to be single-compound-based white-light-emitters (SCWEs) for OLEDs. Though only small proportion of RTP is observed in the photoluminescence (PL) measurement, RTP becomes the major component in electroluminescence (EL) owing to 75% of the charge recombination occurring in the triplet manifolds, leading to the realization of white-light generation by the coexistence of fluorescence and RTP. Combining the results of PL and EL ratiometric emissions for the fluorescence versus phosphorescence, we have then deduced efficiency of ISC and propose the feasibility of white-light electroluminescence by full exploitation of intrinsic fluorescence and phosphorescence based on a single **BINAP** emitter. Overall, the optimized white-light organic light-emitting diodes (WOLEDs) achieve maximum external quantum efficiencies (EQE) of ~1.6%, CE > 3 cd·A^−1^ and CIE 1931 coordinates of (0.37, 0.44). Details of the results and discussion are elaborated in the following sections.

## Results

### Characterization and crystal analysis

To ensure the purity, the three different chiral forms of **BINAP** were sublimed and characterized by ^1^H, ^13^C, ^31^P-NMR (Supplementary Figs. 1–8), and chiral chromatography (Supplementary Figs. 9–13). Figure 1a shows the molecular structure of rac-**BINAP**, R-**BINAP**, and S**-BINAP** adopted from their single cryrstal (See Supplementary Fig. 14 and Supplementary Table 1 for detail). Figure 1b depicts the packing modes of rac-**BINAP**, R-**BINAP**, and S-**BINAP** deduced from single crystal X-ray analysis. Upon careful examination, we notice that the family of **BINAP** crystals belong to the same monoclinic system, but different space group, C2/c, P21, and P21 for rac-**BINAP**, R-**BINAP**, and S-**BINAP**, respectively (Supplementary Table 2). Both rac-**BINAP** and chiral counterparts show similar layered packing but different molecular arrangement. In the unit cell of rac-**BINAP**, a pair of coupled mirror-symmetric molecules, R-**BINAP**, and S-**BINAP**, array in both sides with a stoichiometric ratio of 1:1. As for R-**BINAP** and S-**BINAP**, a rectangular solid crystal was formed by two identical homochiral molecules in a unit cell of pure enantiomers, extending to a sequential layered structure resembling that of rac-**BINAP**. The slightly different molecular packing models in racemism and homochiral analogues play a critical role in adjusting molecular assembly in crystal. The serials of **BINAP** adopt sequential layered conformations with large amounts of intermolecular interactions like C-H···π and π···π (see Fig. 1c, dashed lines). Such attraction forces cause the system to condense into the state of maximum order and ultimately form crystalline^36,37^. For comparison, rac-**BINAP** possesses a much more compact arrangement with the appearance of short interactions between R- and S-configuration. Each molecule is tethered by four adjacent ones in rac-**BINAP** with fourteen short contacts C-H···π (2.870, 2.808, 2.887 Å) and π···π (3.309 Å). However, for the homochiral counterpart, one molecule only interacts with two adjacent ones, having 12 and 10 short contacts in the form of C-H···π, for R-**BINAP**, and S-**BINAP**, respectively. The π···π distances for R-**BINAP** and S-**BINAP** in the same layer are longer than that of rac-**BINAP**, beyond the range of van der Waals interaction (Supplementary Fig. 15). As a result, the crystal density of rac-**BINAP**, S-**BINAP**, and R-**BINAP** are deduced to be 1.276, 1.243, and 1.236 g·cm^−3^ (Supplementary Table 2), respectively.

The more efficient packing in racemic crystal also anticipates its greater stability, as evidenced by the thermogravimetry–differential thermal analysis (TG-DTA) and differential scanning calorimetry (DSC) analyses. Decomposition temperature (5% weight loss, named T_d_) are recorded in the order of rac-**BINAP** (369.5 °C) > S-**BINAP** (366.6 °C) > R-**BINAP** (352.3 °C) (see Supplementary Fig. 16). Also, the glass transition temperatures (*T*_g_) for R-**BINAP** and S-**BINAP** are measured to be 103.08 °C and 103.11 °C, respectively, whereas no *T*_g_ is observed for rac-**BINAP**, indicative of its morphologic stability during the evaporation process. Further support is given by the lack of amorphous-to-crystalline transition (*T*_cr_) for rac-**BINAP**, while it is observable for both S-**BINAP** (157.28 °C) and R-**BINAP** (156.89 °C). Above all, rac-**BINAP** clearly possesses a denser packing model, obeying Wallach’s rule. It is also worth noting that S-**BINAP** has slightly higher density than R-**BINAP** (1.243 versus 1.236 g·cm^−3^). This, on the one hand, may be due to the uncertainty of the third digit after decimal point. On the other hand, because *T*_d_ and *T*_cr_ values of S-**BINAP** are all slightly higher than those of R-**BINAP**, the slightly higher density and stability for S-**BINAP** in crystal form (cf. R-**BINAP**) may not be negligible.

### Photophysical properties

**Fig. 2:**
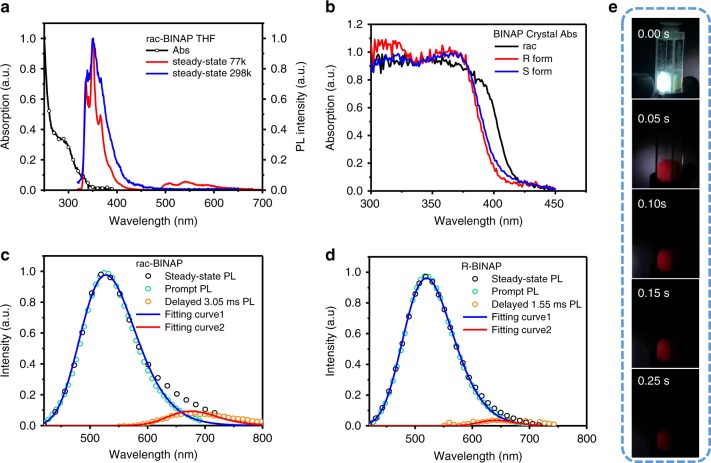
Fundamentally photophysical properties of BINAP. **a** Absorption and emission of rac-**BINAP** in THF. **b** Absorption spectra of the crystal of **BINAP** in each form. **c** The emission of rac-**BINAP** and **d** R-**BINAP** in single crystals. Steady-state emission (black circle) were acquired by continuously 360-nm excitation. The prompt fluorescence (cyan circle) were acquired by pulse excitation (360 nm), and detected by intensified charge-coupled detector (ICCD) opened at *t* = 0 with 50-ns gate width. The phosphorescence (orange circle) were detected by ICCD opened at *t* = 3.05 ms (rac-**BINAP**) and *t* = 1.55 ms (R-**BINAP**) with 200-μs gate width. The fluorescence (blue solid lines) and phosphorescence (red solid lines) spectra were deconvoluted from the steady-state emission with Gaussian function. **e** Photographs of rac-**BINAP** taken under 365-nm UV lamp and after removal of 365-nm UV lamp.

Figure 2a shows the absorpton and emission spectra of rac-**BINAP** in tetrahydrofuran (THF) solution (similar soluton spectra in both R-**BINAP** and S-**BINAP**), while the absorption spectra of rac-**BINAP**, R-**BINAP**, and S-**BINAP** in crystal are shown in Fig. 2b. In comparison, two remarks can be pointed out. First, the red shifted absorption in solid crystal manifests the appreciable C-H···π and π···π interaction observed in the solid crystal (vide supra). Second, the distinct difference in absorption betweem rac-**BINAP** and R- (or S-) **BINAP** in solid supports their different molecular arrangements as elaborated in the aforementioned crystal analyses. Figure 2c and d show the steady-state emission spectra of rac-**BINAP** and R**-BINAP** (see Supplementary Fig. 17a for S-**BINAP**) in their respective single crystal, both of which consist of a major emission in green maximized at ~520 nm.

In addition, for rac-**BINAP**, obviously, an appreciable emission shoulder extended from 600 to 800 nm was observed. Simple spectra deconvolution of the rac-**BINAP** emission renders dual-emission bands maximized at 520 and 680 nm, which are tentatively assinged to fluorescence and phosphorescence, respectively (see Fig. 2c). Upon monitoring at fluorescence (500 nm) and phosphorescence (680 nm) bands, the respective excitation spectra are identical, which are also the same as the absorption spectrum (see Supplementary Fig. 18). The results unambiguously support the same ground-state origin for both emission bands. The intensity ratio for 680-nm emission versus 520-nm emission is ~5.5%, among which the 680-nm RTP can be visualized by the naked eyes upon taking a series of snapshot up to 0.25 s (Fig. 2e). On the other hand, the deconvolution of R-**BINAP** (Fig. 2d) and S-**BINAP** emission spectra (Supplementary Fig. 17a) also resolves a slightly blue-shifted 650-nm emission (cf. 680-nm emission for rac-**BINAP**), and its intensity ratio versus 520-nm emission is significantly less than that of rac-**BINAP** (5.5%), being 1.8% and 2.2%, respectively. The slightly blue-shifted RTP of R- and S-**BINAP** can be referred to the different packing structure and the less intermolecular interaction in thier crystals in comparison with that of rac-**BINAP** (see Fig. 1c; Supplementary Fig. 15). It is also worth of noting that the homochiral R-**BINAP** and S-**BINAP** displayed subtle differences in the spectral shapes of the circularly polarized luminescence (CPL) signals shown in Supplementary Fig. 19 with |g_em_ | ≈(1.46–1.65) × 10^−3^ that were nearly mirror images of each other.

To gain more insights into the photophysical properties, we then used an integrating sphere to measure the absolute emission quantum yield (Q.Y.) of crystals. As a result, the fluorescence and phosphorescence Q.Y. of rac-**BINAP** crystalline were measured to be Φ_f_ = 7.6% and Φ_p_ = 0.42%, respectively. Note that the deduction for each emission is based on the overall emission yield multiplying its intensity ratio. Accordingly, Φ_p_ = 0.42% in PL is considered to be excellent for a persistent organic RTP material of λ ≥ 680 nm (see Supplementary Table 3 and Supplementary Fig. 20), which is commonly subject to dominant vibrational quenching operated by energy gap law^38,39^. The photoluminescence quantum yields of homochiral analogues were also measured, with Φ_f_ of 4.9% and 4.8%, Φ_p_ of 0.09% and 0.11% for R-**BINAP** and S-**BINAP**, respectively. The nearly fourfold Φ_p_ of rac-**BINAP** supports the racemism-enhanced RTP yield.

**Fig. 3:**
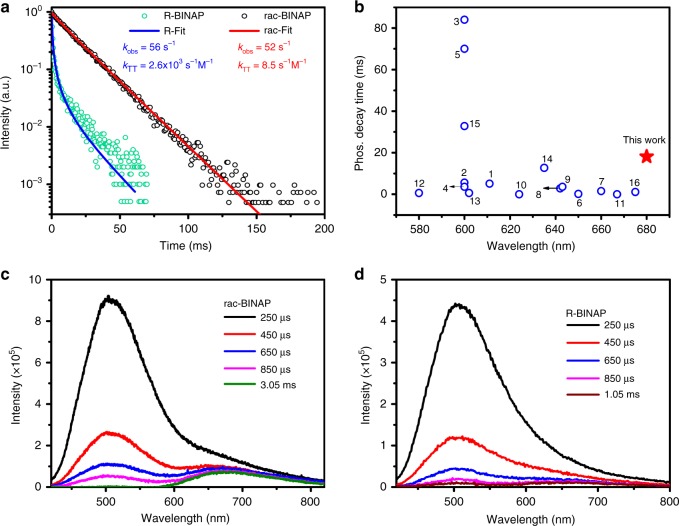
Time-dependently photophysical properties of BINAP. **a** The decay dynamics for the RTP in single crystal of rac-**BINAP** and R-**BINAP**. The rate constants *k*_obs_ and *k*_TT_ (red for rac-**BINAP** and blue for R-**BINAP**) are deduced from the data fit with Eq. (1). **b** The literature reported decay time of orange-red non-metal RTP molecules (for details of each number, please see Supplementary Table 3 and Supplementary Fig. 20)^39–46^. Spectral temporal evolution of the PL spectrum for (**c**) rac-**BINAP** and (**d**) R-**BINAP** in single crystal measured at variant delayed time with a gate width of 200 μs.

Investigated by time-correlated single-photon counting (TCSPC) system, on the one hand, rac-**BINAP** gives a lifetime of 1.4 ns when monitored at ~520-nm emission band (Supplementary Fig. 21), which is unambiguously assigned to the fluorescence nature. On the other hand, R- and S-**BINAP** show slightly shorter lifetime of 0.8 and 0.9 ns, respectively (Supplementary Fig. 21). Further, we had to use a pulsed xenon lamp as the excitation source to resolve the exceedingly long lifetime (>1 ms) of the phosphorescence decay for all **BINAP** series. As a result, the decay profile monitored at 680 nm for rac-**BINAP** and R**-BINAP** is shown in Fig. 3a. The mono exponential like decay of rac-**BINAP** can be fitted with a lifetime of 18.1 ms. For deep-red/near infrared emission, a RTP with ultralong lifetime is rare due to the operation of energy gap law mentioned above. Evidence is provided in Fig. 3b, which shows the RTP decay time for previously reported non-metal organic molecules with orange-red phosphorescence. Apparently, the 680-nm long-lived RTP is record long in terms of peak wavelength and lifetime among the reported non-metal organic RTP molecules.

Under the same pulse-excitation intensity, in contrast to the nearly single-exponential decay of the rac-**BINAP** at 680 nm, the time-resolved signals at 680 nm for R-**BINAP** reveal significant nonlinear behavior, which cannot be well fitted by a single-exponential decay component. This deviation is most plausibly ascribed to the decay of the triplet state (i.e., phosphorescence) subject to TTA. Accordingly, the overall decay dynamics of phosphorescence [P]_*t*_ associated with TTA process can be expressed in Eq. (1)^47^.

1 $$\left[ P \right]_t = \alpha \left[ {T_1} \right]_t = \alpha \frac{{k_{{\mathrm{obs}}}\left[ {T_1} \right]_0}}{{\left( {k_{{\mathrm{obs}}} + k_{{\mathrm{TT}}}\left[ {T_1} \right]_0} \right)e^{k_{{\mathrm{obs}}}t} - k_{{\mathrm{TT}}}\left[ {T_1} \right]_0}}$$

where α is a combination factor of instrument and triplet parameter, such as quantum yield. [T_1_]_*t*_ and [T_1_]_0_ specify the time-dependent T_1_ concentration at time *t* and initial triplet state population (*t*~0), respectively. *k*_obs_ and *k*_TT_ denote the unimolecular decay rate constant and bimolecular decay rate constant for TTA, respectively. Note that *k*_TT_ is concentration-dependent, and we would not solve the exact [T_1_]_0_ here^48^. Due to the same excitation intensity and similar absorption, we assume the same initial triplet-state population in each case. Taking arbitrary α and initially guessed [T_1_]_0_ to be unity, the best curve fit using Eq. (1) gives *k*_obs_ values around 5.6 × 10^1^ s^−1^, and *k*_TT_ values of 2.6 × 10^3^ s^−1^ M^−1^ for R-**BINAP** (Fig. 3a; see Supplementary Fig. 17b for the decay rate of S-**BINAP**). As for the decay rate of rac-**BINAP**, the best fits give similar *k*_obs_ values around 5.2 × 10 s^−1^ but much slower *k*_TT_ values around 8.5 s^−1^ M^−1^, resulting in nearly single-exponential curve (Fig. 3a). The fitted parameters for all **BINAP** series are tabulated in Supplementary Table 4. Since the rac-**BINAP** crystal contains arrangement of molecules with different chirality, TTA is expected to be retarded due to the different neighboring environments that suppresses triplet exciton diffusion. As such, the non-radiative pathway induced from bimolecular TTA is suppressed, leading to the efficient harvest of RTP. Nevertheless, TTA process can be clearly observed in rac-**BINAP** under the high power laser excitation (360 nm) and measured by intensified charge-coupled detector (ICCD), in which the delayed fluorescence maximum at 520 nm populated by TTA mechanism was resolved (see Fig. 3c, d). In theory, the origin of delayed fluorescence [F]_*t*_ should be proportional to [T_1_]_*t*_^2^, and the corresponding decay is virtually half of the lifetime of the phosphorescence^49–51^. To prove this, the decay of delayed fluorescence was probed at 490 nm. As shown in Supplementary Fig. 22, in addition to the irresolvable prompt fluorescence decay, rac-**BINAP** shows a delayed fluorescence with a lifetime fitted to be 8.57 ms, which is approximately half of the time constant of RTP. The results firmly support the occurrence of slight TTA, which may possibly result from the different packing directions under racemic environment. Analogous spectral evolutions are also deduced for R- and S**-BINAP**, and the corresponding data are shown in Fig. 3d and Supplementary Fig. 23, respectively. We also used ICCD gated at *t* = 0 with a window of 50 ns to acquire the prompt fluorescence free from RTP. The prompt fluorescence shown in Fig. 2 is clearly distinct from the steady-state emission by the lack of 680-nm phosphorescence band. In addition, the delayed emissive signals were collected by ICCD with suitable delayed time recorded at *t* = 3.05 ms for rac-**BINAP** and *t* = 1.55 ms for R**-BINAP** with 200-μs gate width, which should be mainly ascribed to the phosphorescence as shown in Fig. 2c, d. Both the fluorescence and phosphorescence show good agreement with the corresponding fitting curves shown in Fig. 2.

To gain more insight into the racemism-enhanced RTP, we attempted to deduce the associated ratiative decay (*k*_p, r_) and non-radiative decay rate constants (*k*_p, nr_) based on ISC efficiency and the intrinsic phosphorescence Q.Y. taken from the EL section (vide infra). Unfortunately, due to the serious TTA for such an exceedingly long RTP even in lower power excitation, it is impossible to deduce phosphorescence radiative decay rate and likewise the ISC for R-**BINAP** and S-**BINAP** forms. For rac-**BINAP**, due to its very small efficiency of TTA, we were able to deduce all relevant parameters under small power excitation. Supplementary Table 5 lists the full parameters of observed decay rate, TTA, ISC efficiency, radiative decay rate, and unimolecular non-radiative decay rate for rac-**BINAP**. Despite the fact that only *k*_obs_ and *k*_TT_ can be obtained for R-**BINAP** and S-**BINAP**, we reemphasize here that the significance as well as impact of the result lies in that under similar *k*_obs_ (see Supplementary Table 5), the TTA rate constant (8.5 s^−1^ M^−1^) of RTP for rac-**BINAP** is more than two orders of magnitude less than that of R-**BINAP** (2.6 × 10^3^ s^−1^ M^−1^) and S-**BINAP** (2.2 × 10^3^ s^−1^ M^−1^). TTA serves as the major deactivation and hence emission quenching channel for R-**BINAP** and S-**BINAP**, while it is drastically reduced in rac-**BINAP**. Evidently, different packing arrangements between rac-**BINAP** and its homochiral analogues play a key role for this prominent discrepancy, in which rac-**BINAP** possesses alternative R and S cross-packing that retards exciton motion and hence greatly suppresses TTA, boosting the emission intensity. This result is intrinsic, which manifests and extends the Wallach’s rule to the chiral–photophysical behavior.

### Electroluminescent (EL) properties

Despite the minor 680-nm RTP in photoluminescence, the more balanced fluorescence versus phosphorescence intensity in the electroluminescence was observed, rendering a white-light emission in both racemic and each homochiral forms. The key difference lies in the small efficiency for ISC (S_1_→T_n_) but high RTP Q.Y., which could not be recognized from the above photoluminescence study (vide infra).

Supplementary Fig. 24 shows PL (*λ*_ex_ = 330 nm) of rac-**BINAP** thin film prepared by vacuum deposition. Clearly, the PL spectrum in solid film also consists of a major peak at ~500 nm, accompanied by a long-wavelength shoulder located at ~600 nm. Similar to that in solid crystal, the emission bands can thus be assigned to fluorescence and phosphorescence, respectively (vide supra). The blue-shifts of both fluorescence and phosphorescence in thin film compared with that in single crystal (520 nm and 680 nm) can be rationalized by the difference of solid morphology^52^, in which **BINAP** has denser packing and hence stronger intermolecular interaction in the single crystal than that in the thin film. Further support of this assignment is confirmed by time-resolved measurement of rac-**BINAP** in the thin film shown in Supplementary Fig. 25 and Supplementary Table 6, in which short (ns) and long decay (ms) components are unambiguously observed upon monitored at 500-nm and 600-nm emission bands, respectively.

**Fig. 4:**
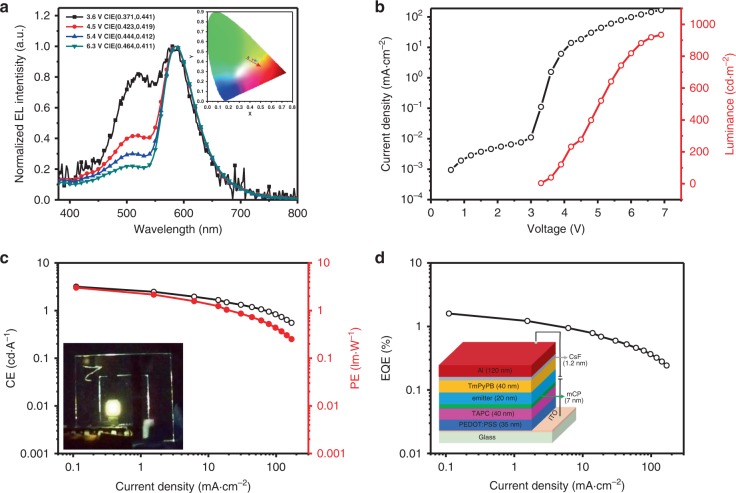
The performance of device 1 based on nondoped rac-BINAP emitter. **a** The EL spectrum under different voltages and insertion of the corresponding CIE coordinates. **b** Current density/luminance as the function of voltage. **c** The CE/PE as the function of current density. Inset: The photograph of **device 1** under electronic excitation. **d** The EQE as the function of current density and insertion of device configuration.

To probe the EL properties, OLEDs were fabricated based on nondoped **BINAP** films with a configuration of [ITO / PEDOT: PSS (35 nm)/TAPC (40 nm)/mCP (7 nm)/nondoped**-**emitter (20 nm, device X)/TmPyPB (40 nm)/CsF (1.2 nm)/Al (120 nm)] as shown in the insertion of Fig. 4d (**device 1**: rac-**BINAP**; **device 2**: R-**NINAP**; **device 3**: S-**BINAP**). Supplementary Fig. 26 illustrates the molecular and device structures accompanied by energy diagram, in which PEDOT: PSS (poly(3,4-ethylenedioxythiophene)-poly(styrenesulfonate)) serves as the hole-injecting layer (HIL) and TAPC (1,1’-bis(di-4-tolylaminophenyl) cyclohexane) acts as the hole-transporting layers (HTL), mCP (1,3-bis(carbazol-9-yl)benzene) is used as the electron-blocking layer (EBL), TmPyPB (1,3,5-tri[(3-pyridyl)-phen-3-yl]benzene) is applied as the electron transporting layer (ETL). The energy levels of HOMO are determined by the onset of the oxidation curve of cyclic voltammogram (Supplementary Fig. 27), while the LUMO levels are deduced by the HOMO and optical absorption band gaps. The HOMO/LUMO of the emitters are almost the same because of the similar molecular structures.

In sharp contrast to the minor phosphorescence intensity in the PL emission, the EL emission shown in Fig. 4a exhibits nearly balanced fluorescence versus phosphorescence intensity at the threshold of the applied voltage of ~3.6 V, and the contribution of phosphorescence increases upon raising the device voltage (Fig. 4a) The balanced emission between fluorescence and phosphorescence indicates that direct population of the triplet state from electron–hole recombination plays a major factor in harvesting RTP in EL. In other words, the low intensity of red phosphorescence in PL should be attributed to the small efficiency of ISC (S_1_→T_n_). Conversely, the population of the triplet state should be statistically 75% in EL, for which the reversed intersystem crossing (RISC: T_1_→S_1_) process is thermally prohibited because of large ΔE_S-T_ (9.7 kcal·mol^−1^) in solid films.

On the basis of difference between the ratio of fluorescence versus phosphorescence intensity for PL and EL (at the threshold voltage), the ISC (S_1_→T_1_) was then calculated to be 9.8% (see Supplementary Discussion). Because the observed fluorescence and phosphorescence yields are 7.6% and 0.42% for rac-**BINAP** in solid, taking 9.8% ISC into account, the intrinsic quantum yields for fluorescence and phosphorescence (Ф_p, intrinsic_) are deduced to be 8.4% and 4.3%, respectively. We then apply these values with initial population upon electrical excitation (25% for singlet exciton, 75% for triplet exciton), and estimate the observed fluorescence and phosphorescence yield to be 2.1% and 3.2%, respectively, for which the ratio of fluorescence versus phosphorescence intensity is close to the EL spectrum (3.6 V), supporting the electroluminescence spectra obtained at 3.6 V (Fig. 4a).

As for the device performance, compared with the other nondoped **devices 2** and **3** based on homochiral analogue emitter (Supplementary Figs. 28, 29; Table 1), **device 1** based on rac-**BINAP** (Fig. 4b–d) displays better performance with a maximum EQE of 1.59%, power efficiency (PE) of 3.03 lm·W^−1^ and current efficiency (CE) of 3.18 cd·A^−1^ with a color-rendering index (CRI) of 73 and Commission International de L’Eclairage (CIE) coordinates of (0.37, 0.44). All the the performances of OLEDs with nondoped **BINAP** as emitters are tabulated in Table 1. To the best of our knowledge, the EQE of 1.59 % is among the highest values reported for the pure organic phosphorescence emitters with similar CIE and CRI ≥ 73 so far (Supplementary Table 7 and Supplementary Fig. 30).

**Table 1:** EL Performances of OLEDs for **BINAP** series.

Device x	V_on_^a^ (V)	L_max_^b^ (cd·m^−2^)	CE_max_^b^ (cd·A^−1^)	PE_max_^b^ (lm·W^−1^)	EQE_max_^b^ (%)	100 cd·m^−2^ (CE/PE/EQE)^c^	CRI^d^	CIE^e^ (*x, y*)
1	3.2	934	3.18	3.03	1.59	2.12/1.73/1.03	73	0.37, 0.44
2	3.2	1026	3.51	3.45	1.58	1.70/1.43/0.78	63	0.45, 0.38
3	3.8	781	3.33	2.37	1.36	3.09/1.99/1.29	73	0.37, 0.37

^a^V_on_ is the turn-on voltage measured at 1 cd·m^−2^.

^b^L_max_ is the maximum luminance (cd·m^−2^); CE_max_ is the maximum current efficiency; PE_max_ is the maximum power efficiency; EQE_max_ is the maximum external quantum efficiency.

^c^the performance of CE, PE, and EQE at 100 cd·m^−2^.

^d^CRI is color-rendering index value.

^e^CIEs are measured at EQE_max_.

To gain in-depth insights into the device performance, the mechanism of applied-voltage-dependent ratiometric emission has drawn our attention. Such voltage-dependent device performance is reversible, and thus cannot be attributed to the chemical decomposition or breakdown of the device structure. This phenomenon somehow disobeys our conventional wisdom because TTA process should be enhanced upon increase of the applied voltage and hence the decrease in the ratio of triplet population. We thus wonder if there exists a path for converting the singlet exciton back to the triplet exciton.

**Fig. 5:**
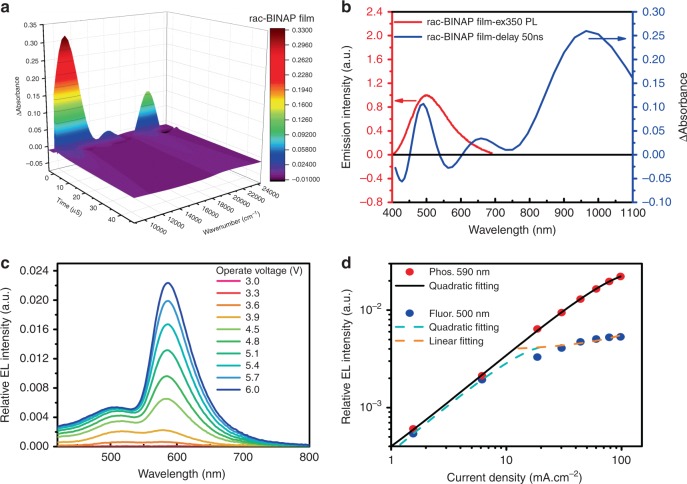
Kinetic analyses of dual emission in BINAP thin film. **a** The side view of the absorbance-wavelength plot for the transient UV–Vis character of rac-**BINAP** in vacuum deposit film on quartz recorded at different delayed time. *λ*_ex_ = 355 nm. **b** The absorbance-wavelength plot for the transient UV–Vis character recorded at 50 ns delayed time of rac-**BINAP** solid film. Also shown is the photoluminescence spectrum (red solid line) of rac-**BINAP** in solid film. **c** Relative EL spectra at different operated voltage. **d** The phosphorescence (Phos., red dot) and fluorescence (Fluor., blue dot) intensity as a function of the operated current density and the corresponding fitting curves.

To verify this viewpoint, we then conducted the nano-microsecond transient absorption measurement for the thin film of rac-**BINAP** with a step-scan Fourier-transform spectrometer^53,54^. The results, depicted in Fig. 5a, b, show a transient absorption signal around 500 nm, which overlaps with part of the fluorescence and can be ascribed to T_1_→T_n_ absorption. Also, the bleaching signals around 430 and 560 nm can be attributed to the stimulated fluorescence (S_1_→S_0_).

The above results suggest a plausible deactivation channel incorporating the singlet–triplet annihilation (STA: S_1_ + T_1_→S_0_ + T_n_), which, together with TTA, influences the ratiometric emission^55^. We then plot the electroluminescence spectra at different operated voltage shown in Fig. 5c. The result clearly indicates that phosphorescence exhibits a continuous growth in intensity as increasing the applied voltage, whereas the increase in the intensity of fluorescence becomes much slower when the operated voltage exceeds 4.8 V. We further plot both EL intensity of phosphorescence and fluorescence as a function of current density (Fig. 5d), so that current-density-dependent emission intensity can be analyzed as shown in Fig. 5d.

Accordingly, it is worth noting that the singlet excitons tend to undergo radiative decay pathway under low operated voltage owing to the much faster radiative decay rate compared with that of triplet excitons. Conversely, under high operated voltage, the singlet excitons can encounter the long-lived triplet excitons more frequently, which fulfills the STA process (S_1_ + T_1_→S_0_ + T_n_), giving back a triplet exciton. To firmly support the statement, we further develop a detailed analysis (see Supplementary Discussion). As a result, the experimental phosphorescence-current-density relationship (Fig. 5d) can be well fitted by a quadratic equation (see Supplementary Eq. (31)), while the fluorescence intensity-current-density plot can be simulated by the sum of a quadratic (see Supplementary Eq. (34)) and a linear (see Supplementary Eq. (36)) equations at lower and higher current density, respectively. To make the complication simple, Supplementary Eqs. (34) and (36) imply that triplet excitons can reach a concentration that STA starts to intervene TTA. At this point, as we continuously increase the voltage, the growth of the fluorescence by TTA process slows down, which is also reflected by the increase of the current density due to STA counter process, leading to the linear response of the fluorescence intensity under high current density.

## Discussion

In summary, both density and stability of racemic rac-**BINAP** single crystal have been proven higher compared with its chiral counterparts, obeying Wallach’s rule. Correlated with this, rac-**BINBP** crystal demonstrates prominent RTP that obviously exhibits higher quantum yield than its homochiral analogues. The kinetic fits clearly suggest that the bimolecular non-radiative quenching TTA is much suppressed in rac-**BINAP** crystals owing to the arrangement of cross chiral counterparts. Accordingly, Wallach’s rule can be extended to the non-metal enhanced, long-lived RTP that is apt to dominant non-radiative quenching and can be rephrased in the case of **BINAP**s to: for the chiral molecules, the solvent-free single crystal of a racemic form is always denser than that of each respective chiral form because of the alternative R- and S- arrangement, which further leads to a suppressed TTA and hence stronger RTP, if there is any. Last but not the least, taking advantage of the results of PL and EL ratiometric emission for the fluorescence versus phosphorescence, we have deduced efficiency of the intersystem crossing and propose the feasibility of white-light generation by exploitation of both fluorescence and RTP based on a single emitter, i.e., **BINAP**. Impressively, the optimized single molecule WOLEDs for rac-**BINAP** achieve a maximum external quantum efficiency (EQE) of ~1.6%, CE > 3 cd·A^-1^ and CIE 1931 coordinates of (0.37, 0.44). The extension of Wallach’s rule to the light emitting chiral chromophores as well as the full exploitation of RTP in WOLEDs paves an avenue to achieve SCWEs via full exploitation of intrinsic fluorescence and phosphorescence.

## Methods

### Reagents and materials

The rac-**BINAP** used in the experiments was purchased from commercial sources (Daicel Chiral Technologies (China) Co., LTD). The rac-**BINAP** was then separated by a chiral column (CHIRALPAK IG (IG00CE-UC011), Hexane/DCM = 40:60 (v/v) to yield R-form (R-**BINAP**) and S form (S-**BINAP**). To ensure the purity, the three forms of **BINAP** were sublimed three times and proved by ^1^H, ^13^C, ^31^P-NMR, and chiral chromatography to be >99% pure. Single crystals for the respective form were then grown in slow evaporation DCM/methanol solution at room temperature.

### Photophysical measurements

Steady-state UV–vis absorption spectra of sample in solution were measured with a Hitachi (U-3310) spectrophotometer. UV–vis absorption spectra and quantum yield of solid sample were recorded with an integrating sphere coupled with Edinburgh FS920 under ambient condition. The nanosecond-scaled lifetime spectra for crystal sample were recorded with an Edinburgh FL 900 single-photon-counting system with a hydrogen-filled lamp as an excitation source. As for the nanosecond-scaled lifetime spectra of sample in thin film, the time-resolved measurements were performed by TCSPC (time-correlated single-photon counting) system (OB-900L Lifetime spectrometer, Edinburgh). The light source was generated from Ti sapphire laser (Tsunami, Spectra Physics, 82 MHz) pulse-selected to reduce its repetition rate to 8.2 MHz, followed by second-harmonic generation to produce excitation beam (360 nm). The polarization of the pump laser was set at the magic angle (54.7˚) with respect to pump beam, in order to eliminate the anisotropy. The temporal resolution is about 20 ps. The millisecond-scale lifetime spectra were carried out on Edinburgh FL920 fluorescence spectrophotometer equipped with microsecond flash-lamp (μF 920 H). The charging voltage of the ICCD was open at different delay times with a tunable gated window synchronized with the firing time of the excitation pulse. In this work, the second harmonic (360 nm, fwhm ~8 ns) of an Nd:YAG laser was used as the excitation pulse. X-ray crystallography was achieved using a Bruker SMART APEX-II CCD diffractometer with graphite monochromated Mo-Kα radiation. Luminescent photographs were recorded using a Cannon EOS 700D single lens digital camera with a hand-held UV lamp switched on and off at room temperature. The CPL spectra were recorded with JASCO CPL-300.

### Step-scan pump–probe time-resolved UV–vis measurements

Step-scan UV–vis spectroscopy: Step-scan UV–vis spectroscopy was performed using a Vertex 80 spectrometer (Bruker) with a Si diode detector. For samples containing pristine **BINAP** on quartz substrates was prepared using a chemical vapour deposition (CVD) technique. In this study, a third harmonic of a Nd:YAG laser (Continuum, Surelite) at 355 nm was used as the pump pulse, which has a duration of 15 ns and a repetition rate of 10 Hz. The background reduction and optimization of pumping power required various combinations of interference filters and were necessary to avoid thermal noise and sample decomposition. A typical excitation energy was adjusted to lower than 200 mJ·cm^−2^. An optimized laser pulse had a decent signal-to-noise ratio to avoid damaging the sample. Moreover, the excitation source was synchronized with the spectrometer and was set at an accurate 45° with respect to the UV–vis probe beam to maximize the pump–probe overlap. In addition, the visible source (tungsten lamp, 24 V, 150 W) was employed as an externally source coupled with a water cooling unit and power supply. During the measurement, the entire UV–vis compartment was purged with 2 bar nitrogen at room temperature.

## Supplementary information

Supplementary Information

## Data Availability

The data that support the findings of this study are available from the corresponding authors upon reasonable request. The X-ray crystallographic coordinates for the compounds releated to Fig. 1 have been deposited at the Cambridge Crystallographic Data Centre (CCDC) under deposition number CCDC 1968817 (rac-**BINAP**), 1968818 (R-**BINAP**), and 1968819 (S-**BINAP**). These data can be obtained free of charge from The Cambridge Crystallographic Data Centre via www.ccdc.cam.ac.
