# Supplementary material for: Exploiting racemism enhanced organic room-temperature phosphorescence to demonstrate Wallach’s rule in the lighting chiral chromophores
Source: Nat Commun. 2020 May 1;11:2145. doi: 10.1038/s41467-020-15976-5 (PMC7195388; doi:10.1038/s41467-020-15976-5)
Supplement: Supplementary file 1 — Supplementary Information [file 41467_2020_15976_MOESM1_ESM.pdf]

Supporting Information

Exploiting Racemism Enhanced Organic Room-Temperature Phosphorescence to Demonstrate Wallach’s Rule in the Lighting Chiral Chromophores

Wu, Huang, Chen et al.

Supplementary Method:

Fabrication of devices.

The materials used in OLED device fabrication were commercially available. To fabricate devices, the ITO-coated glass was used as an anode and sequentially cleaned as standard operation.[1] A 35 nm thick hole injection layer of PEDOT : PSS film was spin-coated on the cleaned ITO substrate and then annealed at 120 °C for 20 min in a vacuum oven. 1,1'-bis(di-4-tolylaminophenyl) cyclohexane (TAPC, 40 nm), 1,3-Bis(carbazol-9-yl)benzene (mCP, 7 nm), BINAP (20 nm), TmPyPB (40 nm), CsF (1.2 nm), and aluminum (120 nm) were continuously deposited using resistive evaporation, respectively. The device area was 0.16 cm^2 for all samples in this work. The thicknesses of spin-coating and evaporation were monitored with a quartz crystal thickness/ratio monitor (model STM-100/MF, Sycon). The J-V-R characteristics were measured using the Keithley 236 system and a calibrated silicon photodiode. Irradiance and luminance were calculated assuming a Lambertian distribution. Electroluminescence (EL) spectra and CIE coordinate were measured using CS-1000 spectoradiometer.

Supplementary Discussion:

Derivations of intrinsic fluorescence and phosphorescence yield.

Under the assumption that the net intersystem crossing yield (x), which includes the effect resulting from triplet-triplet annihilation (TTA), is the same in both photoluminescence (PL) and electroluminescence (EL), x can be calculated from the following equation:

$$\alpha\left(\frac{1-x}{x}\right)=I_F/I_P \tag{1}$$

$$\alpha\left(\frac{25\times(1-x)}{75+25x}\right)=E_F/E_P \tag{2}$$

where α denotes the ratio for radiative rate of fluorescence versus phosphorescence. I_F and I_P denote the PL intensity of fluorescence and phosphorescence, respectively. E_F and E_P denote the EL intensity of fluorescence and phosphorescence. Therefore, x can be solved by dividing eqs 1 with eqs 2. The net intersystem crossing yield (x) can be expressed in following equation:

$$x=\frac{3}{\dfrac{I_F/I_P}{E_F/E_P}-1} \tag{3}$$

The ratio for PL of fluorescence versus phosphorescence is set to be 18.23, which is deduced from the ratio of area from the PL fluorescence and phosphorescence of rac-**BINAP**. As for the ratio for EL of fluorescence versus phosphorescence, the value is set to be 0.58, which is determined from the ratio for area for the EL fluorescence versus phosphorescence under 3.6V operated voltage. Under low operated voltage, TTA is hindered as much as possible. x is thus deduced to be 9.8%. The intrinsic fluorescence and phosphorescence yield can thus be obtained by dividing the respective quantum yield with 1-x and x.

Derivations for the ratio variation of fluorescence versus RTP in Electroluminescence (EL).

In EL mechanism, the energy originates from the migrations of electrons and holes, i.e., hopping between molecules. Thus, we assume that the energy transporting molecules serve as the energy source (ES).[2] On the other hand, molecules undergoing TTA (rac-**BINAP** in this case) serve as annihilators (A). The initial reaction pathways are depicted as follows:

$^{1}ES \xrightarrow{k_{exc}} {}^{1}ES^{*}$	Singlet Exciton excitation (exc)	(R1)
$^{1}ES \xrightarrow{3k_{exc}} {}^{3}ES^{*}$	Triplet Exciton excitation (exc)	(R2)
$^{1}ES^{*} \xrightarrow{k_{qf}} {}^{1}ES$	Singlet Exciton quenching	(R3)
$^{3}ES^{*} \xrightarrow{k_{qp}} {}^{1}ES$	Triplet Exciton quenching	(R4)

Here, k_{exc}, k_{qf}, and k_{qp} denotes the constants of excitation rate, quenching rate of the singlet exciton and triplet exciton, respectively. ^{1}ES, $^{1}ES^{*}$, and $^{3}ES^{*}$ represent the non-excited energy source, singlet exciton, and triplet exciton respectively. Accordingly, the corresponding time-resolved differential equations without any energy transfer processes are depicted below:

$$\frac{d\left[{}^{1}ES\right]}{dt} = k_{qp}\left[{}^{3}ES^{*}\right] + k_{qf}\left[{}^{1}ES^{*}\right] - 4k_{exc}\left[{}^{1}ES\right] \quad (4)$$

$$\frac{d\left[{}^{1}ES^{*}\right]}{dt} = -k_{qf}\left[{}^{1}ES^{*}\right] + k_{exc}\left[{}^{1}ES\right] \quad (5)$$

$$\frac{d\left[{}^{3}ES^{*}\right]}{dt} = -k_{qp}\left[{}^{3}ES^{*}\right] + 3k_{exc}\left[{}^{1}ES\right] \quad (6)$$

Next, we list all the possible energy transfer processes, including the annihilation pathways in triplet-triplet annihilation (TTA) and singlet-triplet annihilation (STA) processes depicted as follows:[2]

$^{1}ES^{*} + {}^{1}A \xrightarrow{k_{SET}} {}^{1}ES + {}^{1}A^{*}$	Singlet Energy Transfer to ground-state annihilator (SET)	(R5)
$^{3}ES^{*} + {}^{1}A \xrightarrow{k_{TET1}} {}^{1}ES + {}^{3}A^{*}$	Primary Triplet Energy Transfer to ground-state annihilator (TET1)	(R6)
$^{3}ES^{*} + {}^{3}A^{*} \xrightarrow{k_{TET2}} {}^{1}ES + {}^{3}A^{**}$	Secondary Triplet Energy Transfer to triplet excited annihilator (TET2)	(R7)
$^{3}A^{*} + {}^{3}A^{*} \xrightarrow{k_{TTA1}} {}^{1}A + {}^{1}A^{*}$	Primary (intermolecular) Triplet-Triplet Annihilation (TTA1)	(R8)
$^{3}A^{**} \xrightarrow{k_{TTA2}} {}^{1}A^{*}$	Secondary (intramolecular) Triplet-Triplet Annihilation (TTA2)	(R9)
$^{1}A^{*} + {}^{3}A^{*} \xrightarrow{k_{STA}} {}^{1}A + {}^{3}A^{**}$	Singlet-Triplet Annihilation (STA)	(R10)
$^{1}A^{*} \xrightarrow{k_{ISC}} {}^{3}A^{*}$	Intersystem crossing process (ISC)	(R11)
$^{1}A^{*} \xrightarrow{k_{FA}} {}^{1}A$	Excited singlet decay of Annihilator (FA)	(R12)
$^{3}A^{*} \xrightarrow{k_{PA}} {}^{1}A$	Excited triplet decay of annihilator (PA)	(R13)

where ^{1}A, $^{1}A^{*}$, $^{3}A^{*}$, and $^{3}A^{**}$ represent the annihilator (A) in its ground-state, first singlet excited-state, first triplet excited-state, and highly triplet excited-state, respectively. k with

subscript is the rate constant of the corresponding reaction list above. Again, the corresponding time-resolved differential equations are listed below:[2]

$$\frac{d\left[{}^{1}ES\right]}{dt}=k_{qp}\left[{}^{3}ES^{*}\right]+k_{qf}\left[{}^{1}ES^{*}\right]+k_{TET1}\left[{}^{3}ES^{*}\right]\left[{}^{1}A\right]+k_{TET2}\left[{}^{3}ES^{*}\right]\left[{}^{3}A^{*}\right]+k_{SET}\left[{}^{1}ES^{*}\right]\left[{}^{1}A\right]-4k_{exc}\left[{}^{1}ES\right] \quad (7)$$

$$\frac{d\left[{}^{1}ES^{*}\right]}{dt}=-k_{qf}\left[{}^{1}ES^{*}\right]-k_{SET}\left[{}^{1}ES^{*}\right]\left[{}^{1}A\right]+k_{exc}\left[{}^{1}ES\right] \quad (8)$$

$$\frac{d\left[{}^{3}ES^{*}\right]}{dt}=-k_{qp}\left[{}^{3}ES^{*}\right]-k_{TET1}\left[{}^{3}ES^{*}\right]\left[{}^{1}A\right]-k_{TET2}\left[{}^{3}ES^{*}\right]\left[{}^{3}A^{*}\right]+3k_{exc}\left[{}^{1}ES\right] \quad (9)$$

$$\frac{d\left[{}^{3}A^{*}\right]}{dt}=k_{TET1}\left[{}^{3}ES^{*}\right]\left[{}^{1}A\right]-2k_{TTA1}\left[{}^{3}A^{*}\right]^{2}-k_{TET2}\left[{}^{3}ES^{*}\right]\left[{}^{3}A^{*}\right]-k_{PA}\left[{}^{3}A^{*}\right]-k_{STA}\left[{}^{1}A^{*}\right]\left[{}^{3}A^{*}\right]+k_{ISC}\left[{}^{1}A^{*}\right] \quad (10)$$

$$\frac{d\left[{}^{3}A^{**}\right]}{dt}=k_{TET2}\left[{}^{3}ES^{*}\right]\left[{}^{3}A^{*}\right]-k_{TTA2}\left[{}^{3}A^{**}\right]+k_{STA}\left[{}^{1}ES^{*}\right]\left[{}^{3}A^{*}\right] \quad (11)$$

$$\frac{d\left[{}^{1}A^{*}\right]}{dt}=k_{SET}\left[{}^{1}ES^{*}\right]\left[{}^{1}A\right]+k_{TTA1}\left[{}^{3}A^{*}\right]^{2}+k_{TTA2}\left[{}^{3}A^{**}\right]-k_{STA}\left[{}^{1}A^{*}\right]\left[{}^{3}A^{*}\right]-k_{ISC}\left[{}^{1}A^{*}\right]-k_{FA}\left[{}^{1}A^{*}\right] \quad (12)$$

$$\frac{d\left[{}^{1}A\right]}{dt}=-k_{SET}\left[{}^{1}ES^{*}\right]\left[{}^{1}A\right]-k_{TET1}\left[{}^{3}ES^{*}\right]\left[{}^{1}A\right]+k_{TTA1}\left[{}^{3}A^{*}\right]^{2}+k_{STA}\left[{}^{1}A^{*}\right]\left[{}^{3}A^{*}\right]+k_{FA}\left[{}^{1}A^{*}\right]+k_{PA}\left[{}^{3}A^{*}\right] \quad (13)$$

We then employ stead-state approximations for the equations list above:

$$0=\left[{}^{1}ES\right]+\left[{}^{1}ES^{*}\right]+\left[{}^{3}ES^{*}\right]-\left[{}^{1}ES\right]_{0} \qquad \text{S balance} \quad (14)$$

$$0=\left[{}^{1}A\right]+\left[{}^{1}A^{*}\right]+\left[{}^{3}A^{*}\right]+\left[{}^{3}A^{**}\right]-\left[{}^{1}A\right]_{0} \qquad \text{A balance} \quad (15)$$

$$0=\frac{d\left[{}^{1}ES^{*}\right]}{dt}=-k_{qf}\left[{}^{1}ES^{*}\right]-k_{SET}\left[{}^{1}ES^{*}\right]\left[{}^{1}A\right]+k_{exc}\left[{}^{1}ES\right] \qquad {}^{1}S^{*} \quad (16)$$

$$0=\frac{d\left[{}^{3}ES^{*}\right]}{dt}=-k_{qp}\left[{}^{3}ES^{*}\right]-k_{TET1}\left[{}^{3}ES^{*}\right]\left[{}^{1}A\right]-k_{TET2}\left[{}^{3}ES^{*}\right]\left[{}^{3}A^{*}\right]+3k_{exc}\left[{}^{1}ES\right] \qquad {}^{3}S^{*} \quad (17)$$

$$0=\frac{d\left[{}^{3}A^{*}\right]}{dt}=k_{TET1}\left[{}^{3}S^{*}\right]\left[{}^{1}A\right]-2k_{TTA1}\left[{}^{3}A^{*}\right]^{2}-k_{TET2}\left[{}^{3}ES^{*}\right]\left[{}^{3}A^{*}\right]-k_{PA}\left[{}^{3}A^{*}\right]-k_{STA}\left[{}^{1}A^{*}\right]\left[{}^{3}A^{*}\right]+k_{ISC}\left[{}^{1}A^{*}\right] \qquad {}^{3}A^{*} \quad (18)$$

$$0=\frac{d\left[{}^{3}A^{**}\right]}{dt}=k_{TET2}\left[{}^{3}ES^{*}\right]\left[{}^{3}A^{*}\right]-k_{TTA2}\left[{}^{3}A^{**}\right]+k_{STA}\left[{}^{1}A^{*}\right]\left[{}^{3}A^{*}\right] \qquad {}^{3}A^{**} \quad (19)$$

$$0=\frac{d\left[{}^{1}A^{*}\right]}{dt}=k_{SET}\left[{}^{1}ES^{*}\right]\left[{}^{1}A\right]+k_{TTA1}\left[{}^{3}A^{*}\right]^{2}+k_{TTA2}\left[{}^{3}A^{**}\right]-k_{STA}\left[{}^{1}A^{*}\right]\left[{}^{3}A^{*}\right]-k_{ISC}\left[{}^{1}A^{*}\right]-k_{FA}\left[{}^{1}A^{*}\right] \qquad {}^{1}A^{*} \quad (20)$$

Followed by the above equations, the derivations are listed below:

From eqs. 16

$$k_{exc}\left[{}^{1}ES\right]=k_{qf}\left[{}^{1}ES^{*}\right]+k_{SET}\left[{}^{1}ES^{*}\right]\left[{}^{1}A\right] \quad (21)$$

From eqs. 17

$$k_{TET1}\left[{}^{3}ES^{*}\right]\left[{}^{1}A\right]=3k_{exc}\left[{}^{1}ES\right]-k_{qp}\left[{}^{3}ES^{*}\right]-k_{TET2}\left[{}^{3}ES^{*}\right]\left[{}^{3}A^{*}\right] \quad (22)$$

From eqs. 18

$$k_{\mathrm{TET2}}\left[{}^{3}ES^{*}\right]\left[{}^{3}A^{*}\right]=k_{\mathrm{TET1}}\left[{}^{3}ES^{*}\right]\left[{}^{1}A\right]-2k_{\mathrm{TTA1}}\left[{}^{3}A^{*}\right]^{2}-k_{\mathrm{PA}}\left[{}^{3}A^{*}\right]-k_{\mathrm{STA}}\left[{}^{1}A^{*}\right]\left[{}^{3}A^{*}\right]+k_{\mathrm{ISC}}\left[{}^{1}A^{*}\right] \quad (23)$$

From eqs. 19

$$k_{\mathrm{TET2}}\left[{}^{3}ES^{*}\right]\left[{}^{3}A^{*}\right]=k_{\mathrm{TTA2}}\left[{}^{3}A^{**}\right]-k_{\mathrm{STA}}\left[{}^{1}A^{*}\right]\left[{}^{3}A^{*}\right] \quad (24)$$

From eqs. 20

$$k_{\mathrm{FA}}\left[{}^{1}A^{*}\right]=k_{\mathrm{SET}}\left[{}^{1}ES^{*}\right]\left[{}^{1}A\right]+k_{\mathrm{TTA1}}\left[{}^{3}A^{*}\right]^{2}+k_{\mathrm{TTA2}}\left[{}^{3}A^{**}\right]-k_{\mathrm{STA}}\left[{}^{1}A^{*}\right]\left[{}^{3}A^{*}\right]-k_{\mathrm{ISC}}\left[{}^{1}A^{*}\right] \quad (25)$$

Combine eqs. 23 with 24

$$k_{\mathrm{TTA2}}\left[{}^{3}A^{**}\right]=k_{\mathrm{TET1}}\left[{}^{3}ES^{*}\right]\left[{}^{1}A\right]-2k_{\mathrm{TTA1}}\left[{}^{3}A^{*}\right]^{2}-k_{\mathrm{PA}}\left[{}^{3}A^{*}\right]+k_{\mathrm{ISC}}\left[{}^{1}A^{*}\right]$$

Then combine with eqs. 22

$$k_{\mathrm{TTA2}}\left[{}^{3}A^{**}\right]=3k_{\mathrm{exc}}\left[{}^{1}ES\right]-k_{\mathrm{qp}}\left[{}^{3}ES^{*}\right]-k_{\mathrm{TET2}}\left[{}^{3}ES^{*}\right]\left[{}^{3}A^{*}\right]-2k_{\mathrm{TTA1}}\left[{}^{3}A^{*}\right]^{2}-k_{\mathrm{PA}}\left[{}^{3}A^{*}\right]+k_{\mathrm{ISC}}\left[{}^{1}A^{*}\right]$$

Then combine with eqs. 24

$$k_{\mathrm{TTA2}}\left[{}^{3}A^{**}\right]=3k_{\mathrm{exc}}\left[{}^{1}ES\right]-k_{\mathrm{qp}}\left[{}^{3}ES^{*}\right]-k_{\mathrm{TTA2}}\left[{}^{3}A^{**}\right]+k_{\mathrm{STA}}\left[{}^{1}A^{*}\right]\left[{}^{3}A^{**}\right]-2k_{\mathrm{TTA1}}\left[{}^{3}A^{*}\right]^{2}-k_{\mathrm{PA}}\left[{}^{3}A^{*}\right]+k_{\mathrm{ISC}}\left[{}^{1}A^{*}\right] \quad (26)$$

Considering 25% of singlet excitons and 75% of triplet excitons in EL system, the overall energy source intensity I_{exc} can be described as:

$$k_{\mathrm{exc}}\left[{}^{1}ES\right]+3k_{\mathrm{exc}}\left[{}^{1}ES\right]=I_{\mathrm{exc}} \quad (27)$$

We then combine eqs. 27 with eqs. 26 to obtain eqs. 28.

$$\frac{3}{4}I_{\mathrm{exc}}-k_{\mathrm{qp}}\left[{}^{3}ES^{*}\right]=2k_{\mathrm{TTA1}}\left[{}^{3}A^{*}\right]^{2}+2k_{\mathrm{TTA2}}\left[{}^{3}A^{**}\right]-k_{\mathrm{STA}}\left[{}^{1}A^{*}\right]\left[{}^{3}A^{**}\right]-k_{\mathrm{ISC}}\left[{}^{1}A^{*}\right]+k_{\mathrm{PA}}\left[{}^{3}A^{*}\right] \quad (28)$$

Further, we define the fluorescence intensity to be I_{F}, which is then combined with eqs. 20.

$$I_{\mathrm{F}}=k_{\mathrm{FA}}\left[{}^{1}A^{*}\right]\phi_{\mathrm{FA}}=\phi_{\mathrm{FA}}\left(k_{\mathrm{TTA1}}\left[{}^{3}A^{*}\right]^{2}+k_{\mathrm{TTA2}}\left[{}^{3}A^{**}\right]+k_{\mathrm{SET}}\left[{}^{1}ES^{*}\right]\left[{}^{1}A\right]-k_{\mathrm{STA}}\left[{}^{1}A^{*}\right]\left[{}^{3}A^{*}\right]-k_{\mathrm{ISC}}\left[{}^{1}A^{*}\right]\right) \quad (29)$$

Next, we define the phosphorescence intensity to be I_{P}

$$I_{\mathrm{P}}=k_{\mathrm{PA}}\left[{}^{3}A^{*}\right]\phi_{\mathrm{PA}}\ ,\quad \left[{}^{3}A^{*}\right]=\frac{I_{\mathrm{P}}}{k_{\mathrm{PA}}\phi_{\mathrm{PA}}} \quad (30)$$

Using eqs. 28, we then obtain the quadratic dependent function for I_{exc} versus I_{P}

$$\frac{3}{4}I_{\mathrm{exc}}-k_{\mathrm{qp}}\left[{}^{3}ES^{*}\right]-2k_{\mathrm{TTA2}}\left[{}^{3}A^{**}\right]+k_{\mathrm{STA}}\left[{}^{1}A^{*}\right]\left[{}^{3}A^{**}\right]+k_{\mathrm{ISC}}\left[{}^{1}A^{*}\right]=2k_{\mathrm{TTA1}}\left(\frac{I_{\mathrm{P}}}{k_{\mathrm{PA}}\phi_{\mathrm{PA}}}\right)^{2}+\frac{I_{\mathrm{P}}}{\phi_{\mathrm{PA}}} \quad (31)$$

In order to simplify the correlation between I_{exc} and I_{F}, we made two assumptions to conduct steady-state approximation. First, under low excitation intensity, TTA should have less effect to the phosphorescence intensity. Accordingly, the following relationship should hold.

$$2k_{\mathrm{TTA1}}\left[{}^{3}A^{*}\right]^{2}+2k_{\mathrm{TTA2}}\left[{}^{3}A^{**}\right]\ll k_{\mathrm{PA}}\left[{}^{3}A^{*}\right]$$

which is applied to eqs. 28, giving eqs. 32.

$$\frac{3}{4}I_{\mathrm{exc}}-k_{\mathrm{qp}}\left[{}^{3}ES^{*}\right]=2k_{\mathrm{TTA1}}\left[{}^{3}A^{*}\right]^{2}+2k_{\mathrm{TTA2}}\left[{}^{3}A^{**}\right]-k_{\mathrm{STA}}\left[{}^{1}A^{*}\right]\left[{}^{3}A^{**}\right]-k_{\mathrm{ISC}}\left[{}^{1}A^{*}\right]+k_{\mathrm{PA}}\left[{}^{3}A^{*}\right]$$

$$\frac{3}{4}I_{\mathrm{exc}}-k_{\mathrm{qp}}\left[{}^{3}ES^{*}\right]+k_{\mathrm{STA}}\left[{}^{1}A^{*}\right]\left[{}^{3}A^{**}\right]+k_{\mathrm{ISC}}\left[{}^{1}A^{*}\right]\approx k_{\mathrm{PA}}\left[{}^{3}A^{*}\right] \tag{32}$$

Therefore

$$k_{\mathrm{TTA1}}\left(\frac{\frac{3}{4}I_{\mathrm{exc}}-k_{\mathrm{qp}}\left[{}^{3}ES^{*}\right]+k_{\mathrm{STA}}\left[{}^{1}A^{*}\right]\left[{}^{3}A^{**}\right]+k_{\mathrm{ISC}}\left[{}^{1}A^{*}\right]}{k_{\mathrm{PA}}}\right)^{2}\approx k_{\mathrm{TTA1}}\left(\frac{k_{\mathrm{PA}}\left[{}^{3}A^{*}\right]}{k_{\mathrm{PA}}}\right)^{2}=k_{\mathrm{TTA1}}\left[{}^{3}A^{*}\right]^{2} \tag{33}$$

Combining eqs 33 with eqs.20

$$k_{\mathrm{FA}}\left[{}^{1}A^{*}\right]=k_{\mathrm{TTA1}}\left[{}^{3}A^{*}\right]^{2}+k_{\mathrm{TTA2}}\left[{}^{3}A^{**}\right]+k_{\mathrm{SET}}\left[{}^{1}S^{*}\right]\left[{}^{1}A\right]-k_{\mathrm{STA}}\left[{}^{1}A^{*}\right]\left[{}^{3}A^{*}\right]-k_{\mathrm{ISC}}\left[{}^{1}A^{*}\right]$$

As a result, a quadratic dependent function for I_{F} can be obtained under low current intensity (eqs. 34):

$$I_{F}=k_{\mathrm{FA}}\left[{}^{1}A^{*}\right]\phi_{\mathrm{FA}}$$
$$=\phi_{\mathrm{FA}}\left(k_{\mathrm{TTA1}}\left(\frac{\frac{3}{4}I_{\mathrm{exc}}-k_{\mathrm{qp}}\left[{}^{3}ES^{*}\right]+k_{\mathrm{STA}}\left[{}^{1}A^{*}\right]\left[{}^{3}A^{**}\right]+k_{\mathrm{ISC}}\left[{}^{1}A^{*}\right]}{k_{\mathrm{PA}}}\right)^{2}+k_{\mathrm{TTA2}}\left[{}^{3}A^{**}\right]+k_{\mathrm{SET}}\left[{}^{1}ES^{*}\right]\left[{}^{1}A\right]-k_{\mathrm{STA}}\left[{}^{1}A^{*}\right]\left[{}^{3}A^{*}\right]-k_{\mathrm{ISC}}\left[{}^{1}A^{*}\right]\right) \tag{34}$$

Second, under high excitation intensity (i.e. high current density), TTA should have large effect to the phosphorescence intensity (annihilation dominant region). Accordingly, the following relationship holds.

$$2k_{\mathrm{TTA1}}\left[{}^{3}A^{*}\right]^{2}+2k_{\mathrm{TTA2}}\left[{}^{3}A^{**}\right]>>k_{\mathrm{PA}}\left[{}^{3}A^{*}\right]$$

which is applied to eqs. 28, giving eqs. 35.

$$\frac{3}{4}I_{\mathrm{exc}}-k_{\mathrm{qp}}\left[{}^{3}ES^{*}\right]=2k_{\mathrm{TTA1}}\left[{}^{3}A^{*}\right]^{2}+2k_{\mathrm{TTA2}}\left[{}^{3}A^{**}\right]-k_{\mathrm{STA}}\left[{}^{1}A^{*}\right]\left[{}^{3}A^{**}\right]-k_{\mathrm{ISC}}\left[{}^{1}A^{*}\right]+k_{\mathrm{PA}}\left[{}^{3}A^{*}\right]$$

$$\frac{3}{4}I_{\mathrm{exc}}-k_{\mathrm{qp}}\left[{}^{3}ES^{*}\right]+k_{\mathrm{STA}}\left[{}^{1}A^{*}\right]\left[{}^{3}A^{**}\right]+k_{\mathrm{ISC}}\left[{}^{1}A^{*}\right]\approx 2k_{\mathrm{TTA1}}\left[{}^{3}A^{*}\right]^{2}+2k_{\mathrm{TTA2}}\left[{}^{3}A^{**}\right] \tag{35}$$

Gather the above eqs. 35 and eqs. 20, a linear dependent function for fluorescence intensity (I_{F}) can be obtained under high current density.

$$I_{\mathrm{F}}=k_{\mathrm{FA}}\left[{}^{1}A^{*}\right]\phi_{\mathrm{FA}}\approx\phi_{\mathrm{FA}}\left(\frac{\frac{3}{4}I_{\mathrm{exc}}-k_{\mathrm{qp}}\left[{}^{3}ES^{*}\right]-k_{\mathrm{STA}}\left[{}^{1}A^{*}\right]\left[{}^{3}A^{**}\right]-k_{\mathrm{ISC}}\left[{}^{1}A^{*}\right]}{2}+k_{\mathrm{SET}}\left[{}^{1}ES^{*}\right]\left[{}^{1}A\right]\right) \tag{36}$$

Eqs. 31, 34 and 36 are used as the fitting functions to the experimental data shown in **Fig. 5d** in the main text. The fitting procedures were performed by Origin 2016.

As described above, strictly speaking, the "Quadratic" (eqs. 34) and "Linear" (eqs. 36) components of fluorescence and "Quadratic" phosphorescence (eqs. 31) intensities are not pure linear and quadratic character because of the existence of ${}^{3}ES^{*}$ and ${}^{1}ES^{*}$, which are proportional to the excitation intensity.

Supportive data for Characterization.

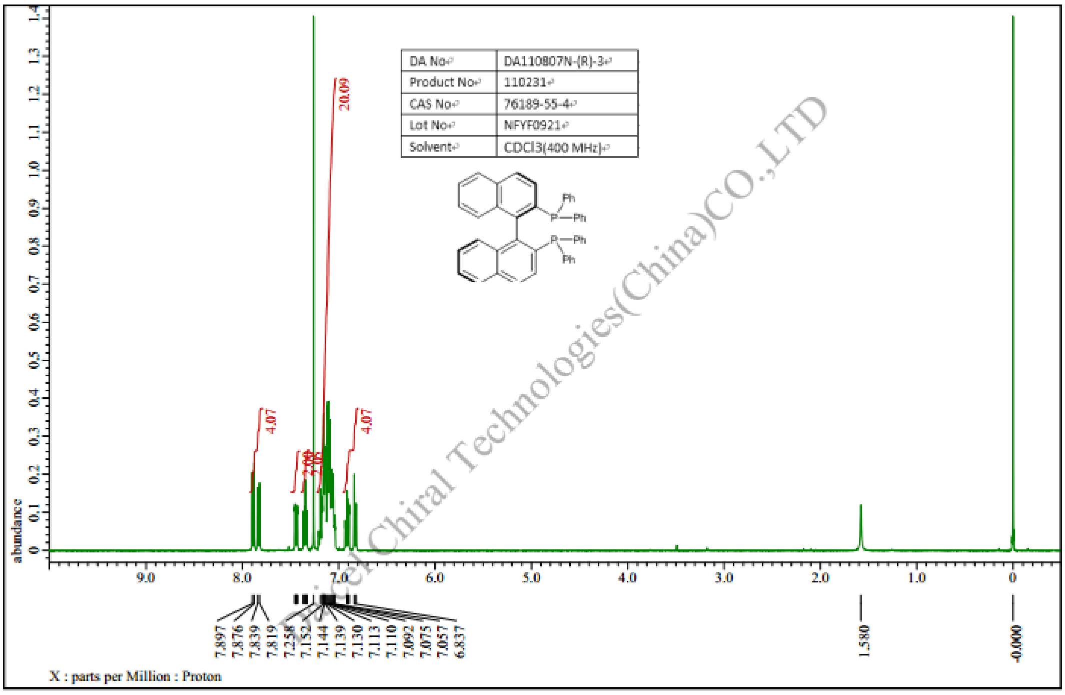

Supplementary Figure 1. ^{1}H NMR of R-**BINAP**.

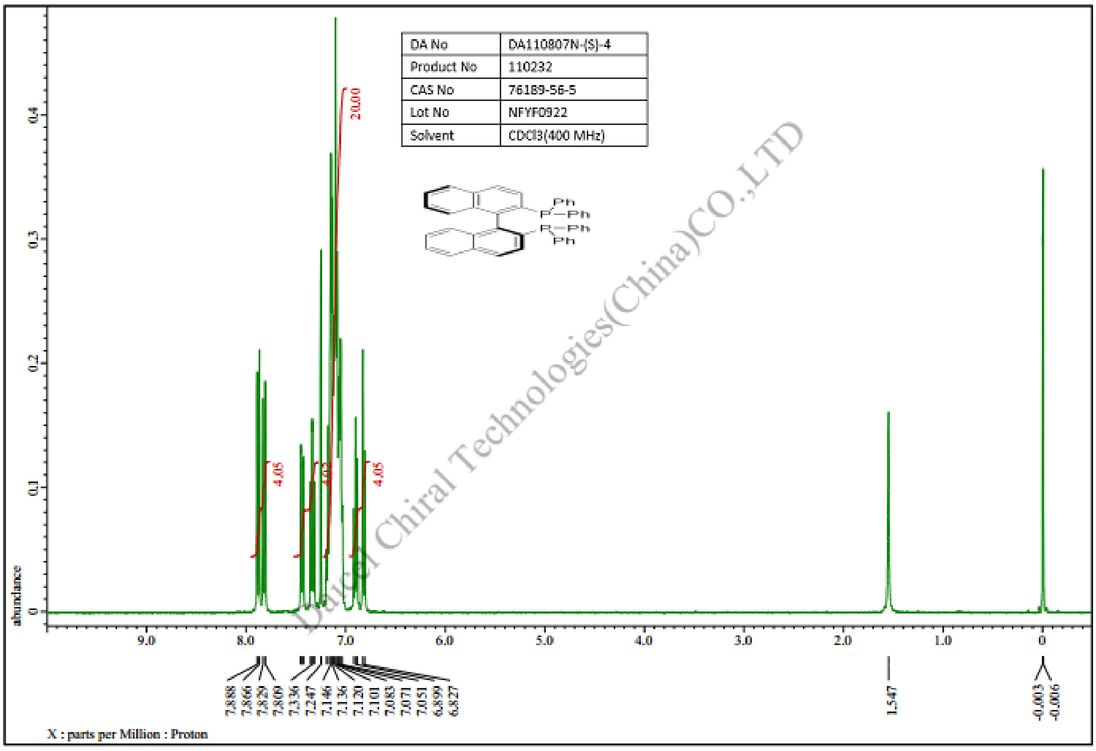

Supplementary Figure 2. ^{1}H NMR of S-**BINAP**.

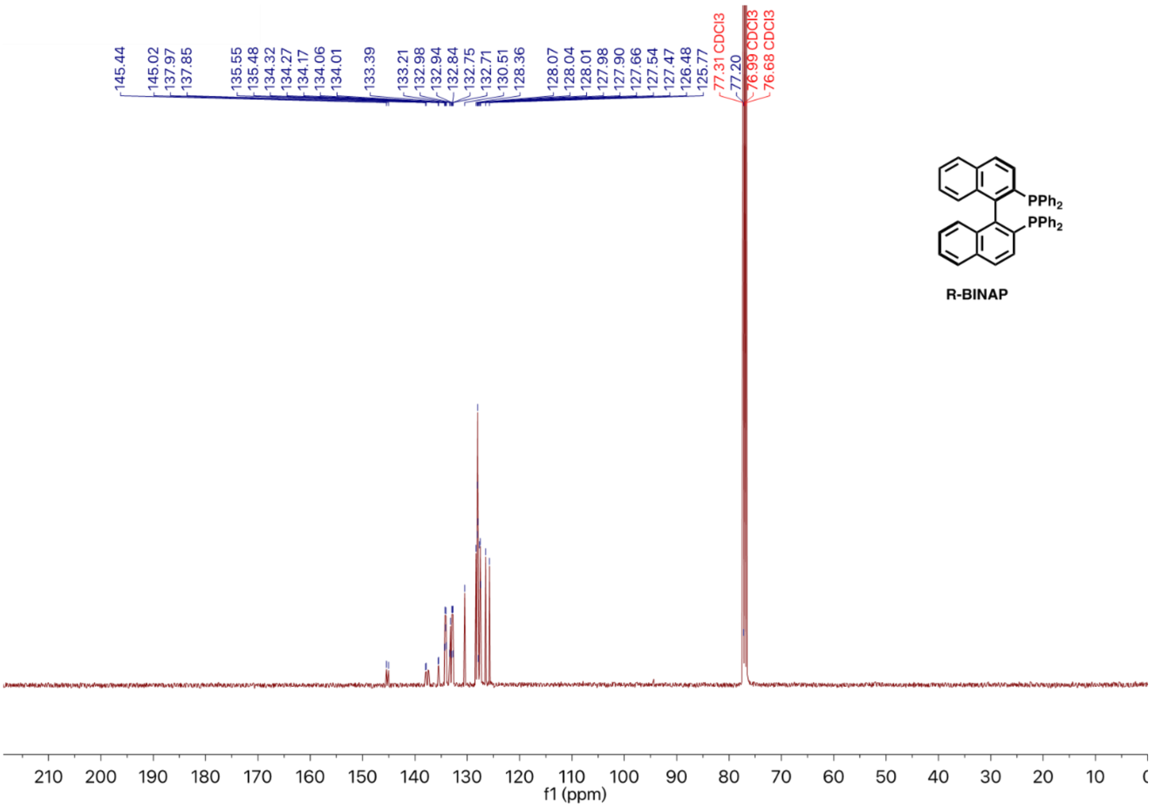

Supplementary Figure 3. ^{13}C NMR of R-**BINAP** in $CDCl_3$.

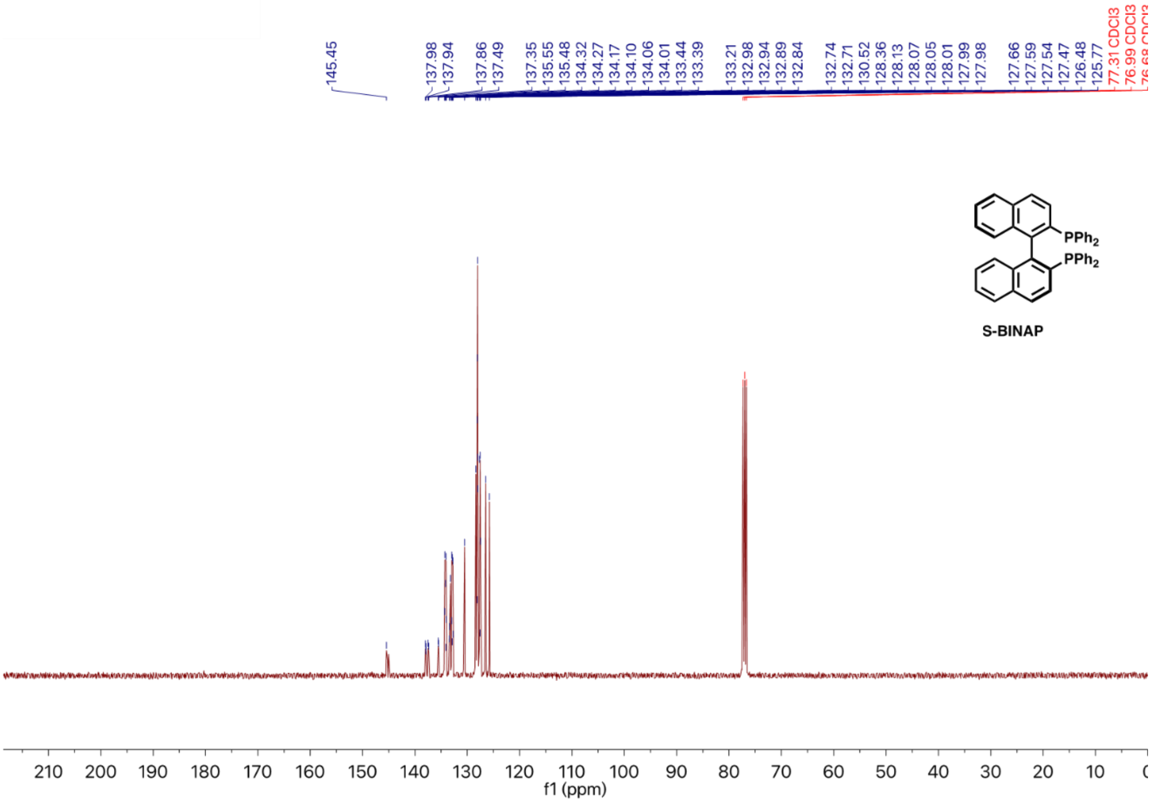

Supplementary Figure 4. ^{13}C NMR of S-**BINAP** in $CDCl_3$.

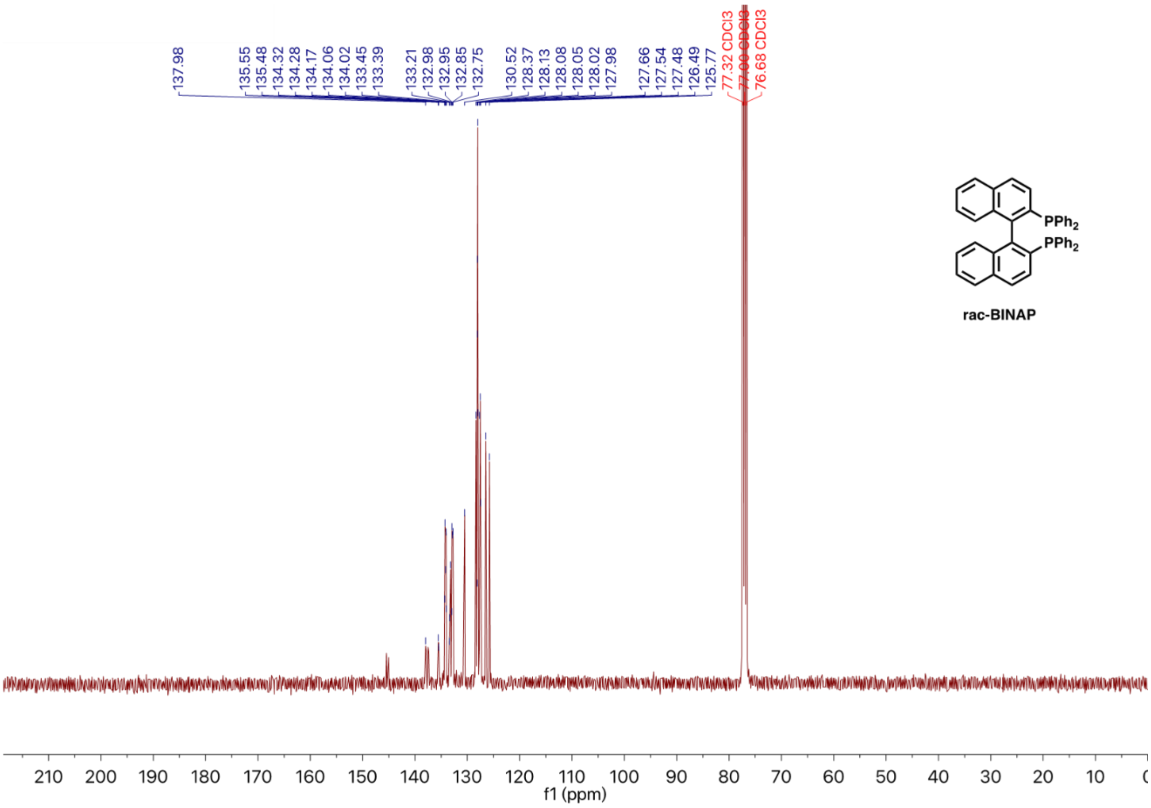

Supplementary Figure 5. ^{13}C NMR of rac-**BINAP** in $CDCl_3$.

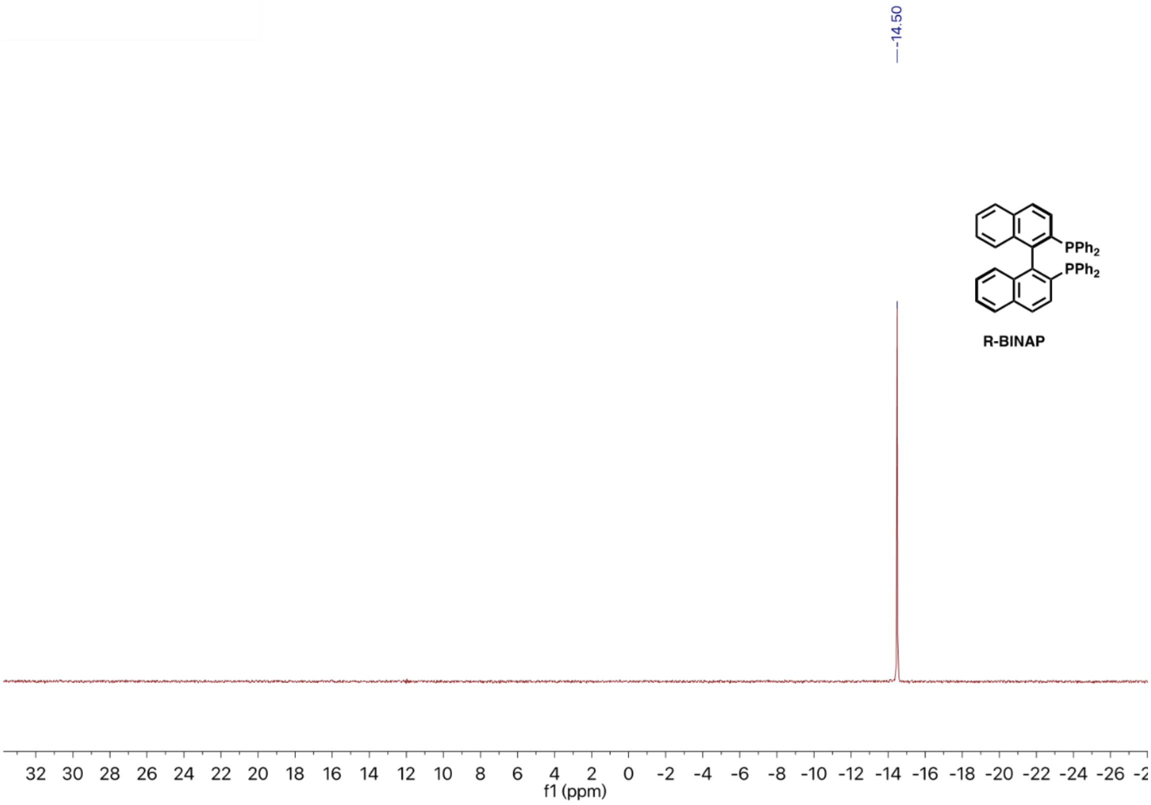

Supplementary Figure 6. ^{31}P NMR of R-**BINAP** in $CDCl_3$.

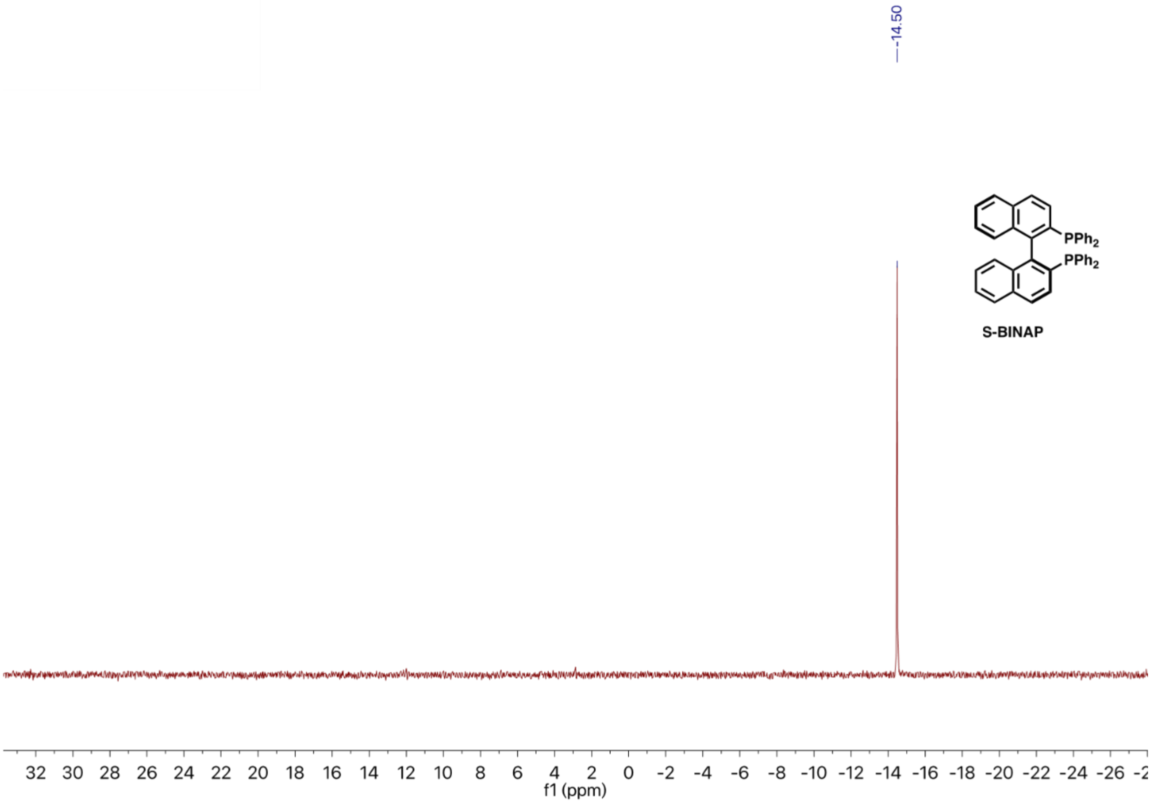

Supplementary Figure 7. ^{31}P NMR of S-**BINAP** in $CDCl_3$.

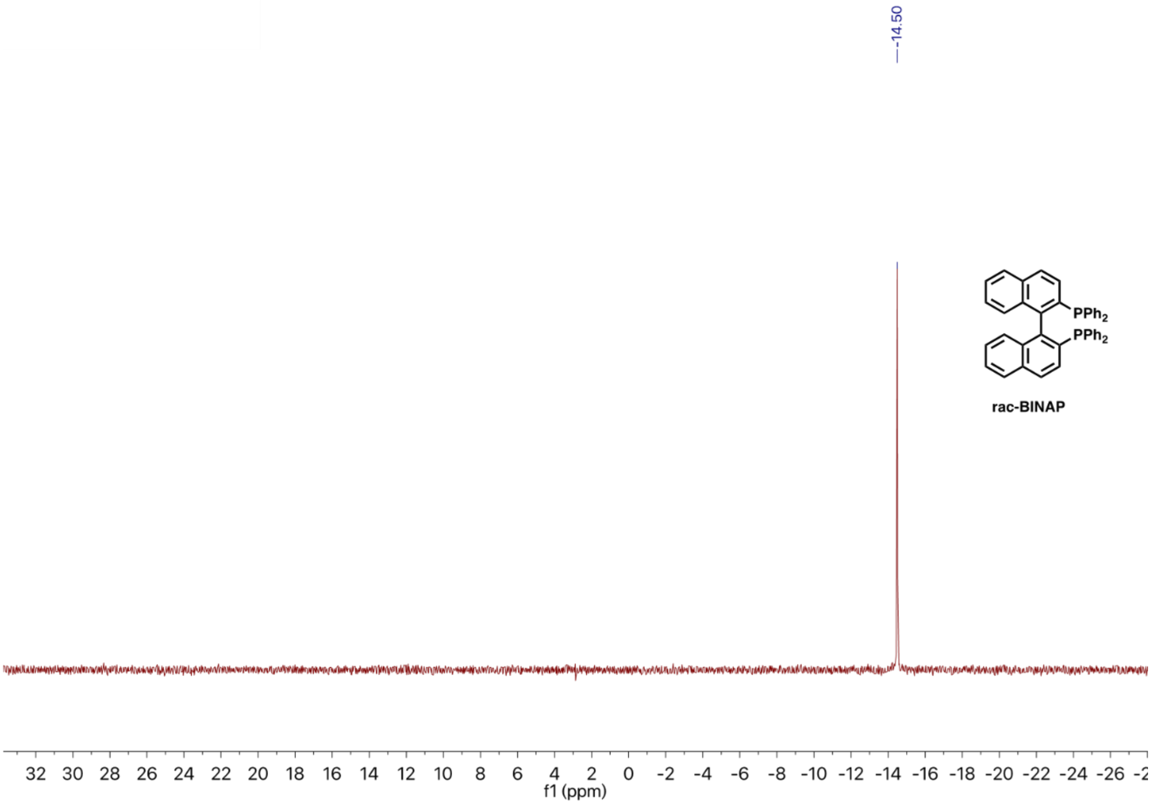

Supplementary Figure 8. ^{31}P NMR of rac-**BINAP** in $CDCl_3$.

CHEMICAL ANALYSIS TEST REPORT

Product Name:	1,1'-[(1R)-[1,1'-binaphthalene]-2,2'-diyl]bis[1,1-diphenylphosphine]
Product No.:	110231
Cas No.:	76189-55-4
Lot No.:	NFYF0921

Column	:	Welch Xtiamte C18 0.46 cm I.D. ×15 cm L, 5 μm
Injection	:	2 μ l
Mobile phase	:	B：MeOH(0.05%NH3) (v/v)
Gradient		NA
Flow rate	:	1.0ml/min
Wave length	:	UV 254nm
Temperature	:	30℃
Sample solution	:	2.0 mg/ml in DCM20%M80%
HPLC equipment	:	Shimadzu UPLC 30A QA&QC-HPLC-11

<Chromatogram>

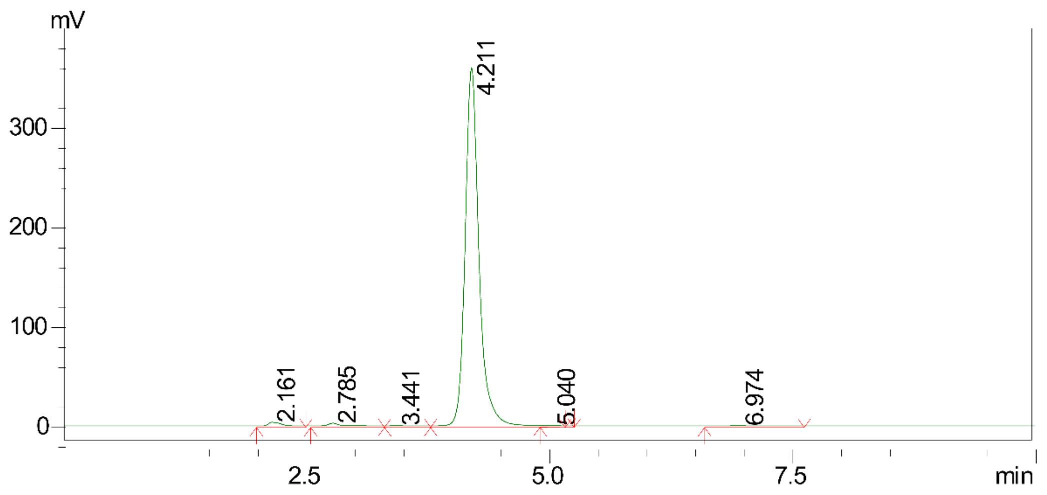

<Peak Table>

Peak No.	Time	Area	Area %	Plate number	Tailing	Resolution
1	2.161	28775	0.801	1505	1.669	--
2	2.785	20272	0.565	3530	1.353	3.042
3	3.441	2129	0.059	2612	--	2.873
4	4.211	3535876	98.465	4441	1.249	2.949
5	5.040	431	0.012	7781	0.932	3.444
6	6.974	3498	0.097	3230	1.437	5.377

Supplementary Figure 9. HPLC of R-**BINAP**.

CHEMICAL ANALYSIS TEST REPORT

Product Name:	1,1'-[(1S)-[1,1'-binaphthalene]-2,2'-diyl]bis[1,1-diphenylphosphine]
Product No.:	110232
Cas No.:	76189-56-5
Lot No.:	NFYF0922

Column	:	Welch Xtimate C18 0.46 cm I.D. ×15 cm L, 5 μm
Injection	:	2 μl
Mobile phase	:	MeOH(0.05%NH3)
Flow rate	:	1.0ml/min
Wave length	:	UV254nm
Temperature	:	30℃
Sample solution	:	1.0 mg/ml in $CHCl_3$10%ACN90%
HPLC equipment	:	Shimadzu UPLC30AD QA&QC-HPLC-11

<Chromatogram>--Column SN#411502347

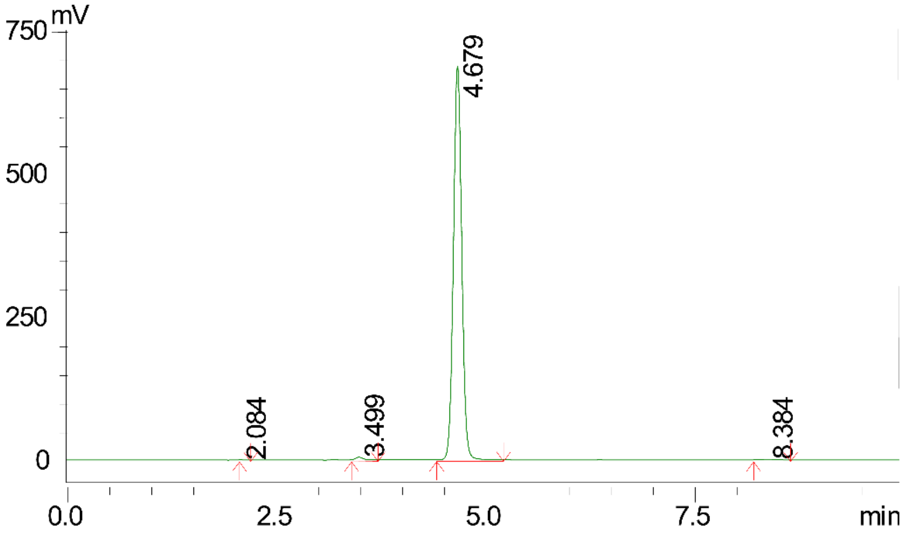

<Peak Table>

Peak No.	Time	Area	Area %	Plate number	Tailing	Resolution
1	2.084	481	0.010	6176	1.704	--
2	3.499	26459	0.551	7193	1.208	10.437
3	4.679	4761511	99.205	9412	1.043	6.593
4	8.384	11229	0.234	11286	1.145	14.568

Supplementary Figure 10. HPLC of S-**BINAP**.

Chiral Analysis report for 1，1′-[[1，1′-binaphthalene]-2，2′-diyl]bis[1，1-diphenylphosphine]

DAICEL-SMP-16 J_1

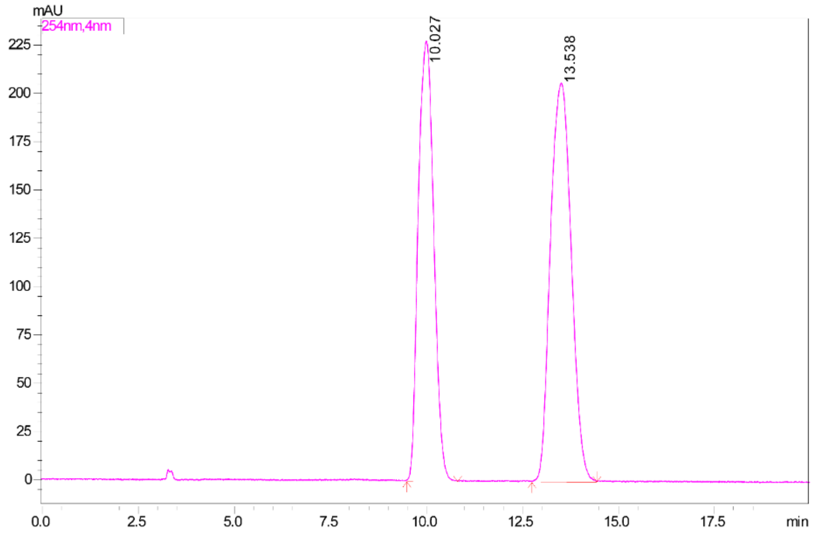

Peak No.	Time	Area	Area %	Plate number	Tailing	Resolution
1	10.027	6399262	45.199	3076	1.083	--
2	13.538	7758726	54.801	3082	1.069	4.135

Supplementary Figure 11. Chiral Analysis report of rac-**BINAP**.

Chiral Analysis report for
1,1'-[(1R)-[1,1'-binaphthalene]-2,2'-diyl]bis[1,1-diphenylphosphine]

Product Name:	1,1'-[(1R)-[1,1'-binaphthalene]-2,2'-diyl]bis[1,1-diphenylphosphine]

Remark:

Original product	1,1'-[(1R)-[1,1'-binaphthalene]-2,2'-diyl]bis[1,1-diphenylphosphine]
Product No.:	110231
Cas No.:	76189-55-4
Lot No.:	NFYF0921

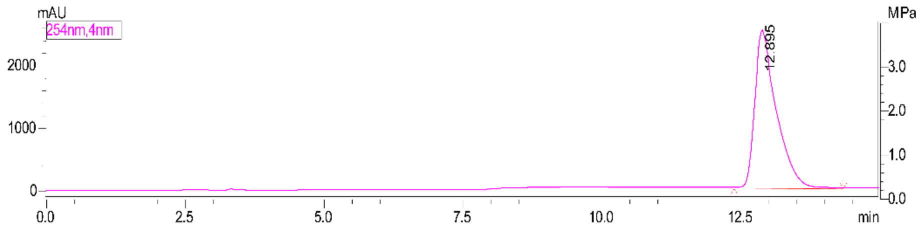

Peak No.	Time	Area	Area %	Plate number	Tailing	Resolution
1	12.895	66421499	100.000	8118	1.709	--

Supplementary Figure 12. Chiral Analysis report of R-**BINAP**.

Chiral Analysis report for
1,1'-[(1S)-[1,1'-binaphthalene]-2,2'-diyl]bis[1,1-diphenylphosphine]

Product Name:	1,1'-[(1S)-[1,1'-binaphthalene]-2,2'-diyl]bis[1,1-diphenylphosphine]

Remark:

Original product	1,1'-[(1S)-[1,1'-binaphthalene]-2,2'-diyl]bis[1,1-diphenylphosphine]
Product No.:	110232
Cas No.:	76189-56-5
Lot No.:	NFYF0922

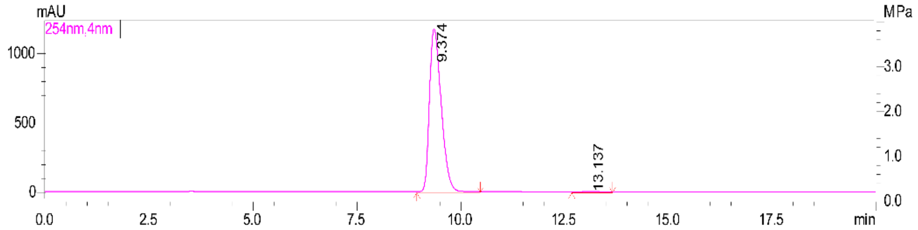

Peak No.	Time	Area	Area %	Plate number	Tailing	Resolution
1	9.374	22905002	99.856	5165	1.424	--
2	13.137	32917	0.144	5992	1.099	6.267

Supplementary Figure 13. Chiral Analysis report of S-**BINAP**.

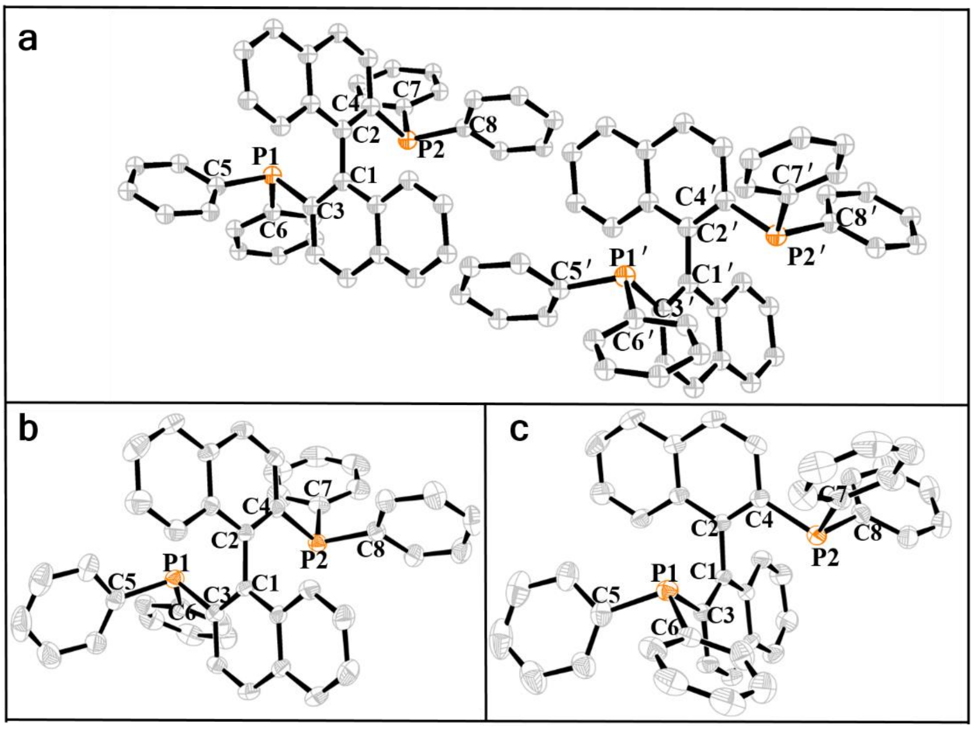

Supplementary Figure 14. The crystal geometry of (a) rac-**BINAP**, (b) R-**BINAP**, and (c) S-**BINAP** depicted with thermal ellipsoids shown at 50% (hydrogen atoms are omitted for clarity). Note that in rac-**BINAP**, the left molecule is R-form, and the right one is S-form with atom number in corresponding order. As for the rac-**BINAP** crystal**,** it is worth to note that R- and S-**BINAP** show the same structure parameters except for its chirality in the racemic environment. However, both R- and S-**BINAP** in racemic environment show different values with respect to their homochiral crystal (see **Table S1** in detail).

Supplementary Table 1. Selected bond lengths [Å] and angles [°] of **BINAP**

**parameters**		rac-**BINAP**[a]	R-**BINAP**	S-**BINAP**
**bond lengths (Å)**	C1-C2	1.499	1.508	1.496
	P1-C3	1.839	1.851	1.836
	P2-C4	1.839	1.841	1.848
	P1-C5	1.833	1.840	1.830
	P1-C6	1.836	1.843	1.831
	P2-C7	1.836	1.831	1.833
	P2-C8	1.833	1.839	1.834
**angles (°)**	C1-C3-P1	118.79	118.79	118.17
	C2-C4-P2	118.79	118.35	118.48
	C3-P1-C5	102.77	102.18	101.76
	C3-P1-C6	100.83	100.37	103.27
	C4- P2-C7	100.83	103.57	100.35
	C4- P2-C8	102.77	102.07	101.79
**torsion (°)**	C4- C2-C1-C3	93.31	91.74	91.66

[a] All the corresponding parameters of R-/S-form is identical to each other in rac-**BINAP**, and the items listed is represented by R-form.

Supplementary Table 2. Structure data of rac-**BINAP**, R- **BINAP**, and S- **BINAP** single crystals.

Name	rac-**BINAP**	R-**BINAP**	S-**BINAP**
Formula	$C_{44}H_{32}P_2$	$C_{44}H_{32}P_2$	$C_{44}H_{32}P_2$
Temperature/K	173	173	173
Crystal system	monoclinic	monoclinic	monoclinic
Space group	C2/c	$P2_1$	$P2_1$
	a= 19.492(3)	a= 9.130(3)	a= 9.1246(10)
Cell Lengths (Å)	b= 9.1725(13)	b= 18.817(5)	b= 18.7595(18)
	c= 19.047(3)	c= 9.997(3)	c= 9.9821(10)
	α= 90	α= 90	α= 90
Cell Angles (o)	β= 107.888(3)	β= 103.090(7)	β= 103.180(3)
	γ= 90	γ= 90	γ= 90
Cell Volume ($Å^3$)	3240.8(8)	1673.0(8)	1663.7(3)
Z	4	2	2
Density (g/cm^3)	1.276	1.236	1.243
F(000)	1304.0	652.0	652.0
Crystal size (mm^3)	0.28 × 0.15 × 0.11	0.19 × 0.15 × 0.12	0.6 × 0.25 × 0.12
Radiation	MoKα (λ = 0.71073)	MoKα (λ = 0.71073)	MoKα (λ = 0.71073)
CCDC number	1968817	1968818	1968819

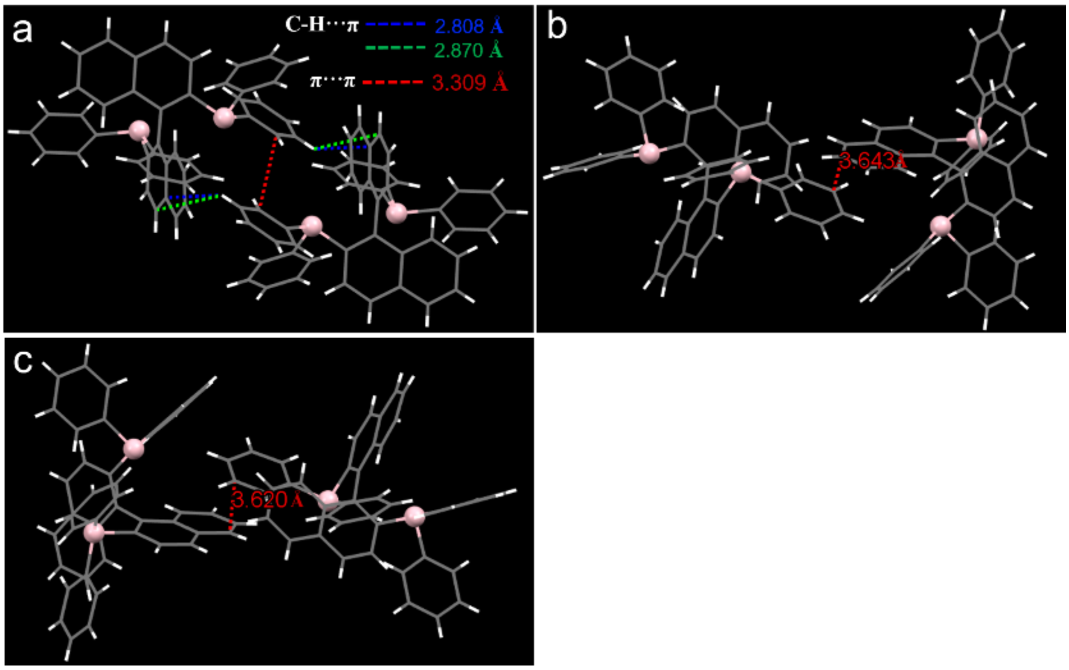

Supplementary Figure 15 Short contacts between the two molecules of the same layer in (a) rac-**BINAP**, (b) R-**BINAP** and (c) S-**BINAP**.

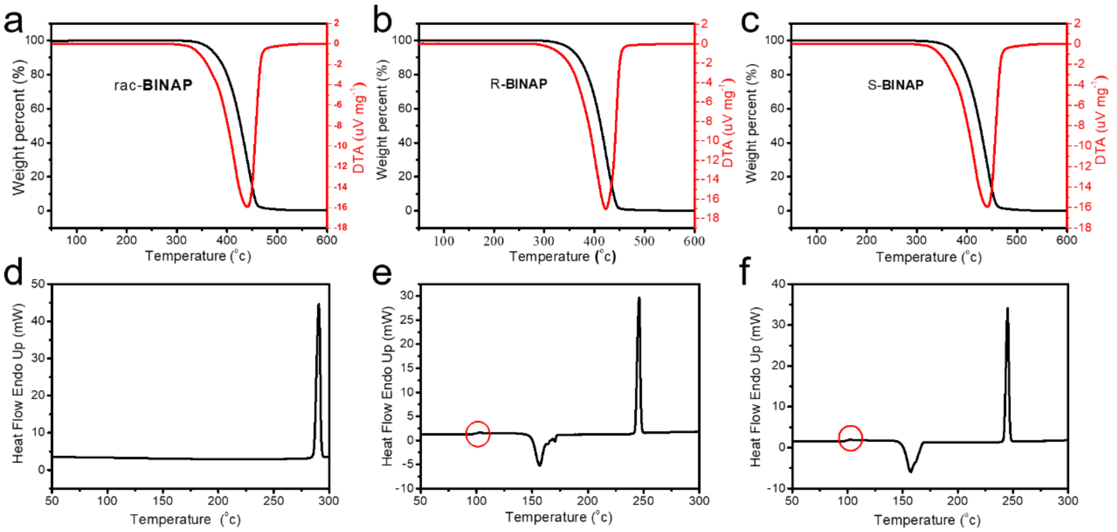

Supplementary Figure 16. TGA curves and differential scanning calorimetry (DSC) analysis of (a,d) rac-**BINAP**, (b,e) R-**BINAP**, and (c,f) S-**BINAP**.

Supportive data for Photophysical properties.

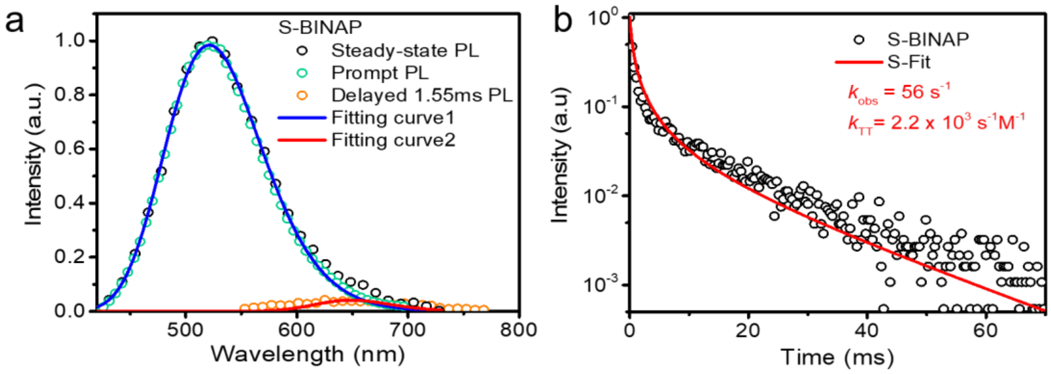

Supplementary Figure 17. (a) The emission of S-**BINAP** in single crystals: (black circle) steady-state emission acquired by cw 360 nm excitation, (cyan circle) the prompt fluorescence acquired by pulse excitation (360 nm) and ICCD opened at t=0 with 50 ns gate width, (blue and red line) the spectrum deconvolution of the steady state emission. (orange circle) The phosphorescence detected by ICCD opened at t = 1.55 ms (S-**BINAP**) with 200 μs gate width. (b) The decay dynamics of the RTP of R-**BINAP**. The rate constant k_{obs} and k_{TT} are deduced from the fitting with eq. 1.

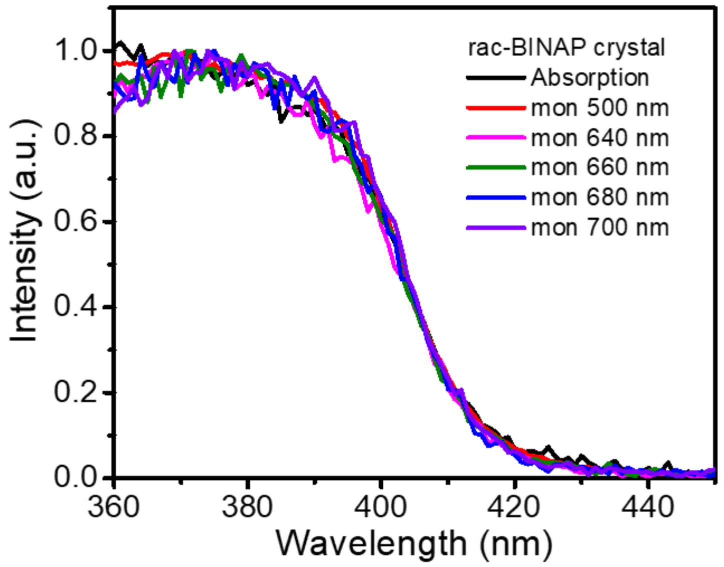

Supplementary Figure 18. The excitation spectra of rac-**BINAP** in crystal.

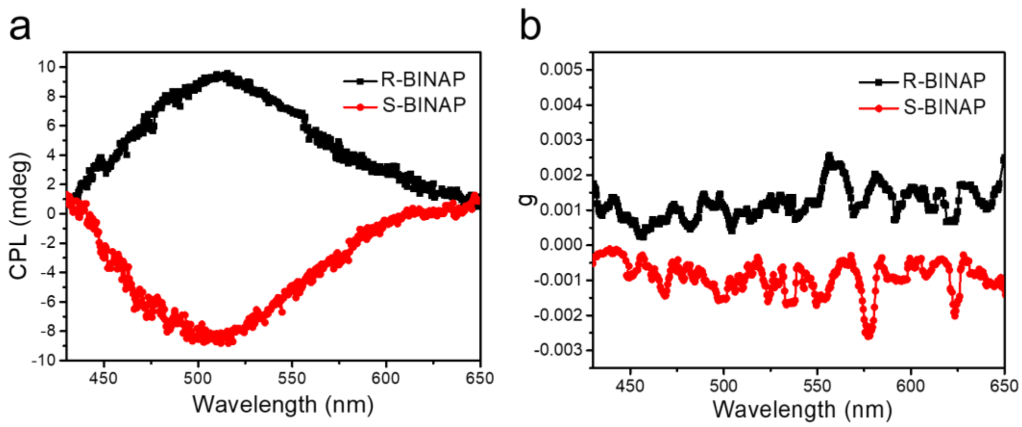

Supplementary Figure 19. R-**BINAP** and S-**BINAP** displayed subtle differences in the spectral shapes of the CPL signals (particularly, at extreme wavelengths around 500 nm) with $|g_{em}|$≈(1.46-1.65)×10^{-3} that were nearly mirror images of each other.

Supplementary Table 3. Photophysical properties of the reported metal-free red phosphors from literature

Compound	λ_{em} (nm)	Φ (%)	τ_p (ms)	Reference
1	611	0.05	5.1	[3]
2	600	0.05	5.6	[3]
3	600	0.0057	84	[3]
4	600	0.046	3.6	[3]
5	600	0.021	70	[3]
6	650	0.5	0.15	[4]
7	660	-	1.5	[4]
8	642	2.8	0.55	[5]
9	643	3.6	0.59	[5]
10	624	5.4	0.7	[5]
11	667	3.4	0.5	[5]
12	580	1	0.6	[6]
13	602	14.6	0.605	[7]
14	635	12.5	12.7	[8]
15	600	6.1	32.8	[9]
16	675	21.7	1.1	[10]
rac-**BINAP**	680	0.42	18.1	This work

Note that for structures of the molecules list in literature are shown in **Supplementary Figure 20** below.

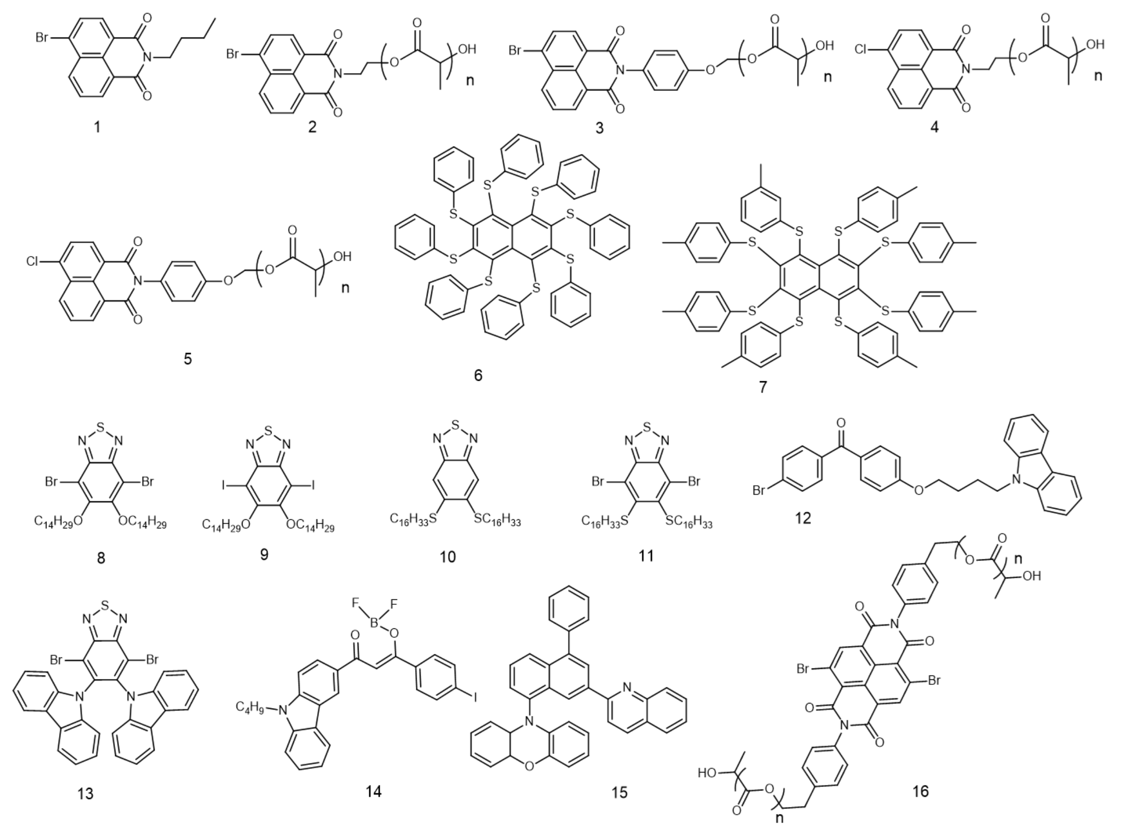

Supplementary Figure 20. The reported molecular structures with red phosphorescence from literature (see **Supplementary Table 3**)

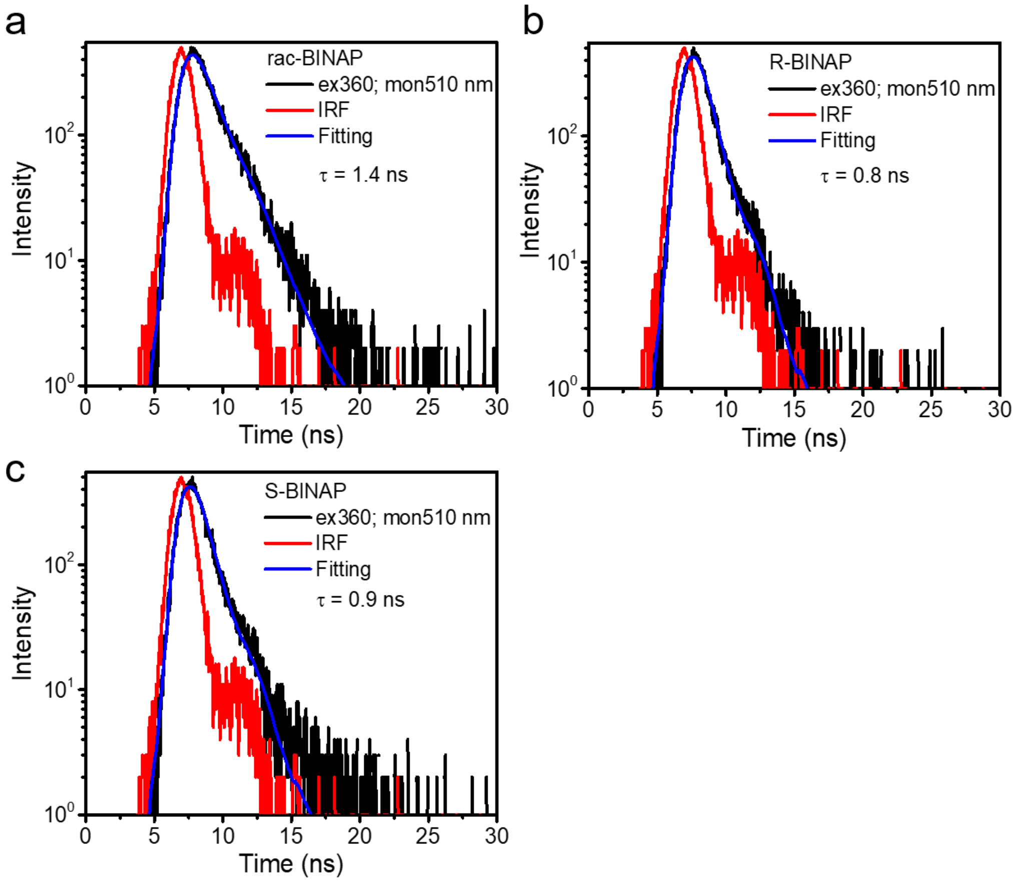

Supplementary Figure 21 Lifetime measurement for the fluorescence of crystal **BINAP** series.

Supplementary Table 4. Fitting Parameters of Dynamic of **BINAP** Series.

Crystal	Delayed Fluorescence		Phosphorescence		α	$[T_1]_0$	k_{obs}	k_{TT}
	Short /μs[a]	Long /ms	Short /μs[a]	Long /ms	(a.u.)	(M)	(s^{-1})	($s^{-1}M^{-1}$)
rac-**BINAP**	(0.996)[b] 598	(0.004)[b] 8.57	--	18.1	0.96	1	52	8.5
S-**BINAP**	(0.992)[b] 553	(0.008)[b] 4.92	(0.896)[b] 616	(0.104)[b] 12.0	1.02	1	56	2200
R-**BINAP**	(0.991)[b] 531	(0.009)[b] 4.61	(0.913)[b] 705	(0.087)[b] 11.3	1.04	1	56	2600

[a] The short component of lifetime denotes the component fitted to be among hundreds of μs.

[b] The number in the parentheses denotes the pre-exponential factor.

Supplementary Table 5. Photophysical properties of RTP from **BINAP** series.

Title Molecules	k_{obs} (s^{-1})[a]	k_{TT} ($s^{-1}M^{-1}$)[a]	ISC efficiency (%)	$\Phi_{p,\ intrinsic}$ (%)	$k_{p,\ r}$ (s^{-1})[b]	$k_{p,\ nr}$ (s^{-1})[c]
rac-**BINAP**	52	8.5	9.8	4.3	2.2	50
R-**BINAP**	56	2600	NA	NA	NA	NA
S-**BINAP**	56	2200	NA	NA	NA	NA

Note that NA denotes "not available".

a) Rate constants deduced from fit shown **Fig. 3a** and **Supplementary Figure 17b**.

b) The radiative rate ($k_{p,\ r}$) is calculated by $k_{p,\ r} = \Phi_{p,\ intrinsic} \times k_{obs}$.

c) The non-radiative rate ($k_{p,\ nr}$) is calculated by $k_{obs} - k_{p,\ r}$.

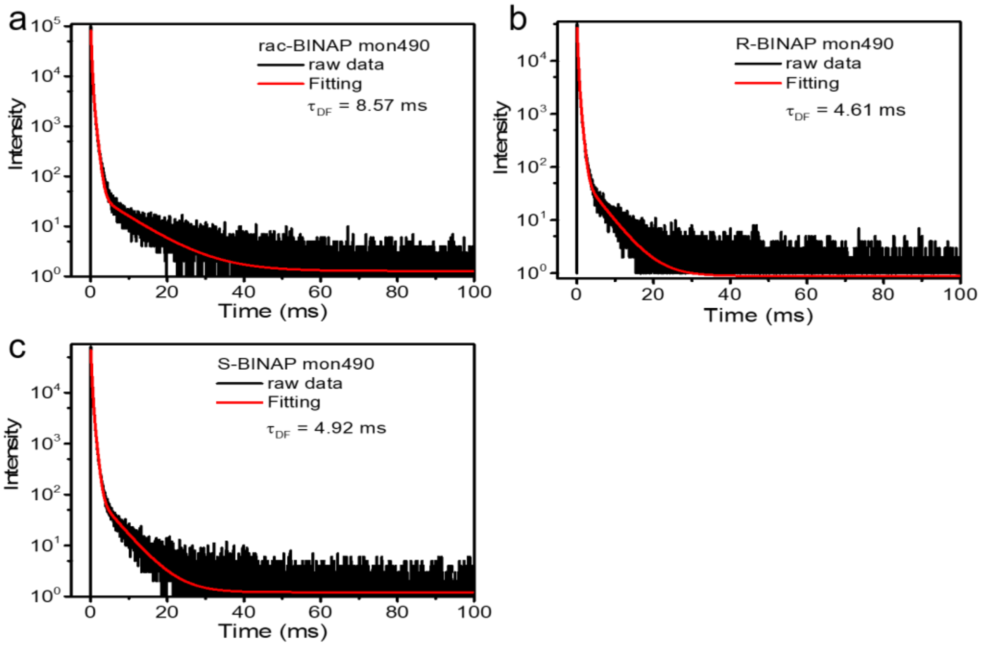

Supplementary Figure 22. Lifetime measurements for the delayed fluorescence of crystal **BINAP** series. Note that for all **BINAP** series, the fast decay component was irresolvable and was attributed to the signal of fluorescence of **BINAP**. Please see **Supplementary Table 4** for the fitting results.

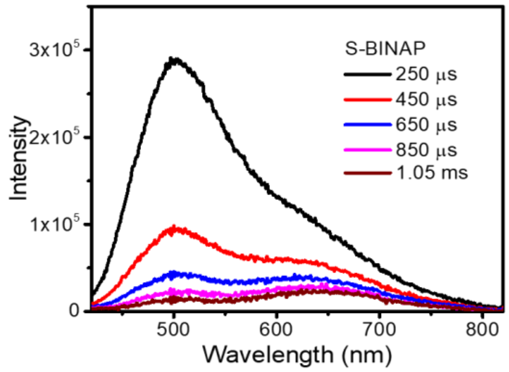

Supplementary Figure 23. The temporal evolution of both RTP and delayed fluorescence in single crystals at denoted time delay acquired by intensified charge coupled detector (ICCD) with 360 nm ND:YAG for excitation. The gate width was set to be 200 μs for each acquisition time.

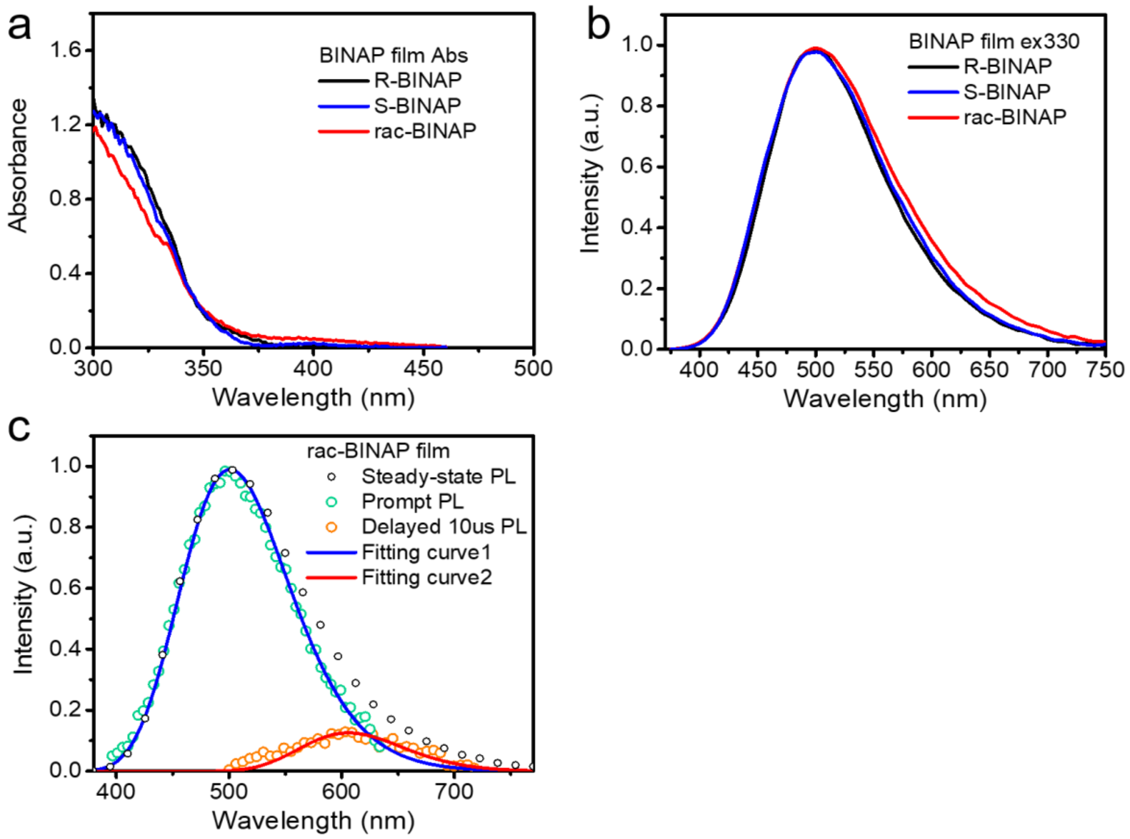

Supplementary Figure 24. Steady-state (a) absorption and (b) PL spectra (λ_{ex} = 300 nm) of **BINAP** series in thin film. (c) The emission of rac-**BINAP** in thin film: (black circle) steady-state emission acquired by continuously 360 nm excitation. (cyan circle) The prompt fluorescence acquired by pulse excitation (360 nm) and detected by intensified charge coupled detector (ICCD) opened at t=0 with 50 ns gate width. (orange circle) The phosphorescence detected by ICCD opened at t = 10 μs with gate width of 100 μs. (blue and red solid lines) The fluorescence (blue) and phosphorescence (red) spectra convoluted from the steady state emission.

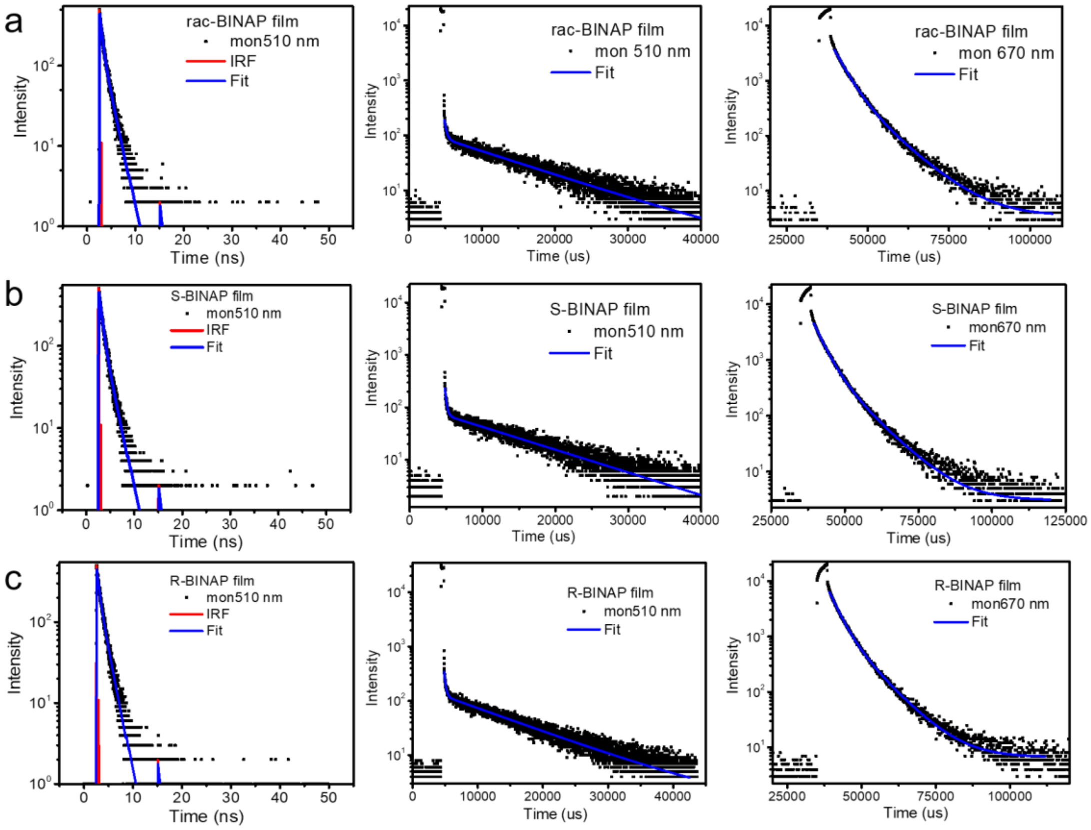

Supplementary Figure 25 Lifetime measurements for (a) rac-**BINAP**, (b) S-**BINAP**, and (c) R-**BINAP** in thin film at 298 K. Figures in the left are the nanosecond-scaled lifetime measurements (λ_{ex} = 360 nm), while figures in the middle and right are micro- to millisecond-scale lifetime measurement (λ_{ex} = 376 nm).

Comparing with the population decay of RTP measured in single crystals, all the decay curves of **BINAP**s measured in thin films show similar temporal evolution. This can be rationalized by the amorphous arrangement of **BINAP**s in thin films, while the crystals of **BINAP**s possess specific and ordered packing structure, resulting in the different population decay for the racemic versus homochiral crystals.

Supplementary Table 6. The summary of lifetime of **BINAP** series in thin film.

compound	$\tau_{510\ nm}$ (short)	τ_{510nm} (long)	τ_{670nm}
rac-**BINAP**	τ_1 = 0.41 ns<br>τ_2 = 1.48 ns	τ_1 = 0.28 ms<br>τ_2 = 10.00ms	τ_1 =3.47 ms<br>τ_2 = 8.64 ms
S-**BINAP**	τ_1 = 0.52 ns<br>τ_2 = 1.51 ns	τ_1 = 0.21 ms<br>τ_2 = 9.98 ms	τ_1 = 3.33 ms<br>τ_2 = 8.64 ms
R-**BINAP**	τ_1 = 0.41 ns<br>τ_2 = 1.37 ns	τ_1 = 0.23 ms<br>τ_2 = 10.01 ms	τ_1 = 3.13 ms<br>τ_2 = 8.03 ms

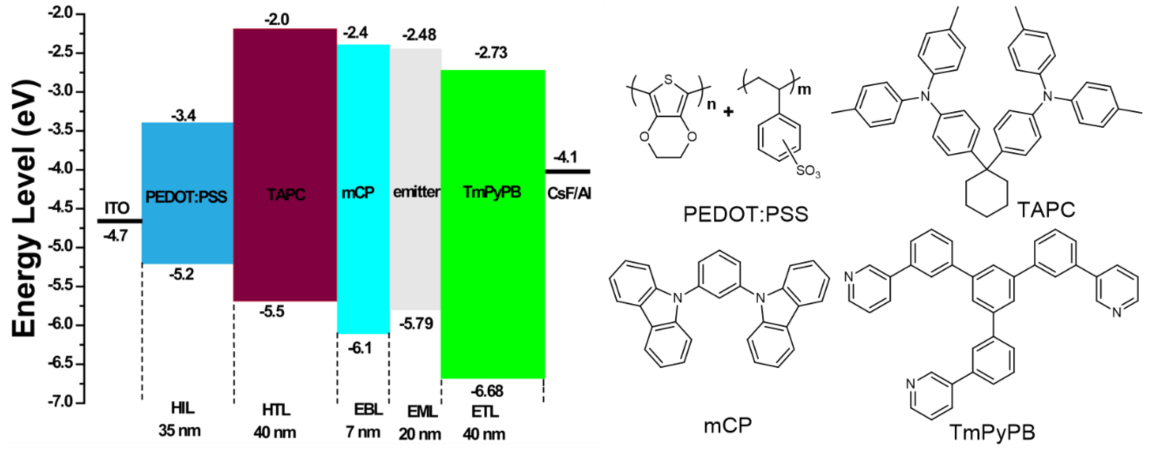

Supplementary Figure 26. The energy level diagram and molecular structures for materials utilized for **BINAP** device fabrication in this study.

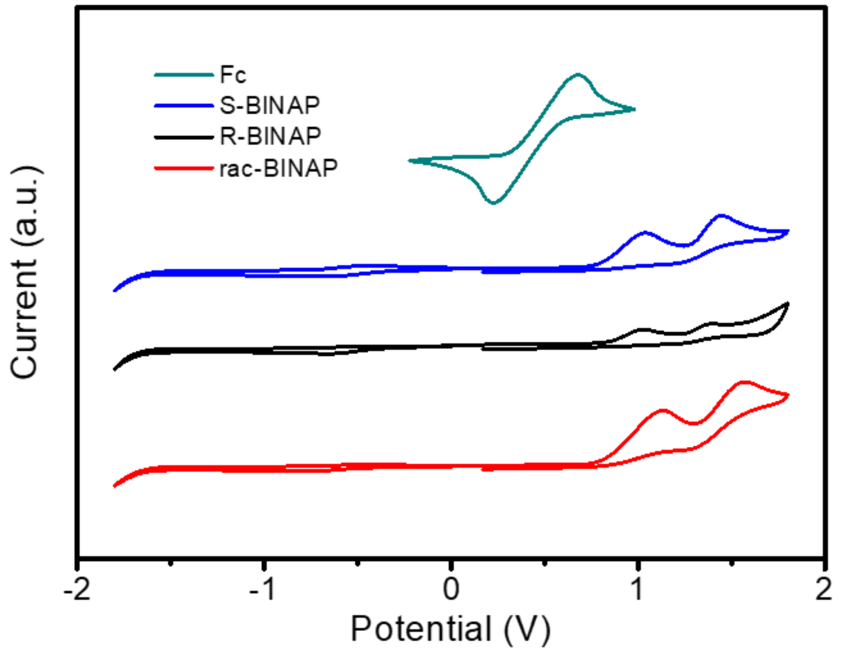

Supplementary Figure 27. Cyclic voltammogram for **BINAP** series. The energy levels of HOMO are determined by the onset of the oxidation curves of cyclic voltammogram (HOMO = -[E_{OX}-$E_{(Fc/Fc+)}$+4.8] eV = -[1.44-0.45+4.8]= -5.79 eV); the energy gaps are then deduced from the optical absorption onset recorded at 375 nm (3.31 eV). (LUMO = -2.48 eV).

Supportive Figures for Devices Characterization.

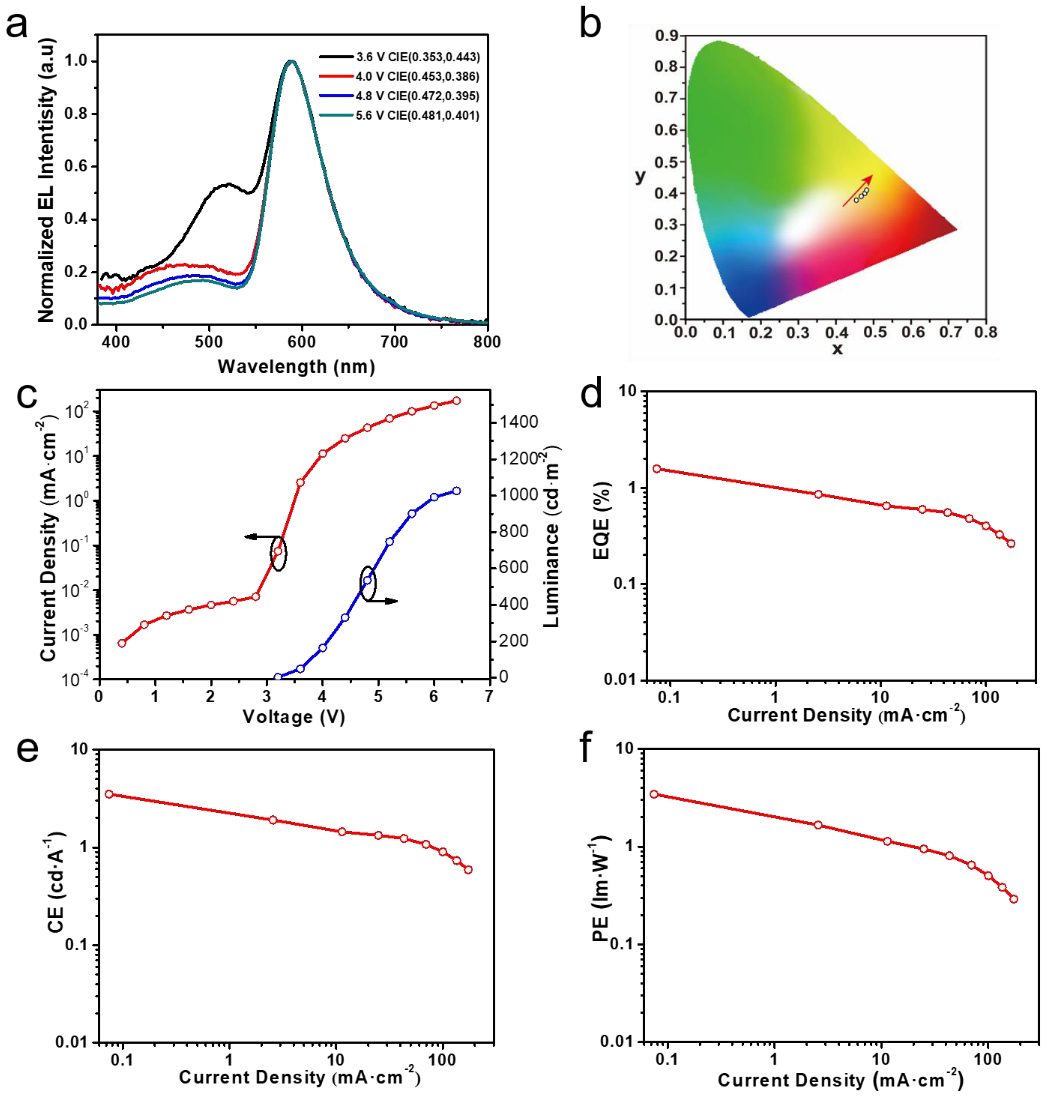

Supplementary Figure 28. Electroluminescence performance of device 2 based on **R-BINAP**: (a) EL spectrum under different operated voltages; (b) The CIE coordinates; (c) current density-voltage-luminance curve; (d) external quantum efficiency- current density curve; (e) current efficiency - current density curve; (f) power efficiency - current density curve.

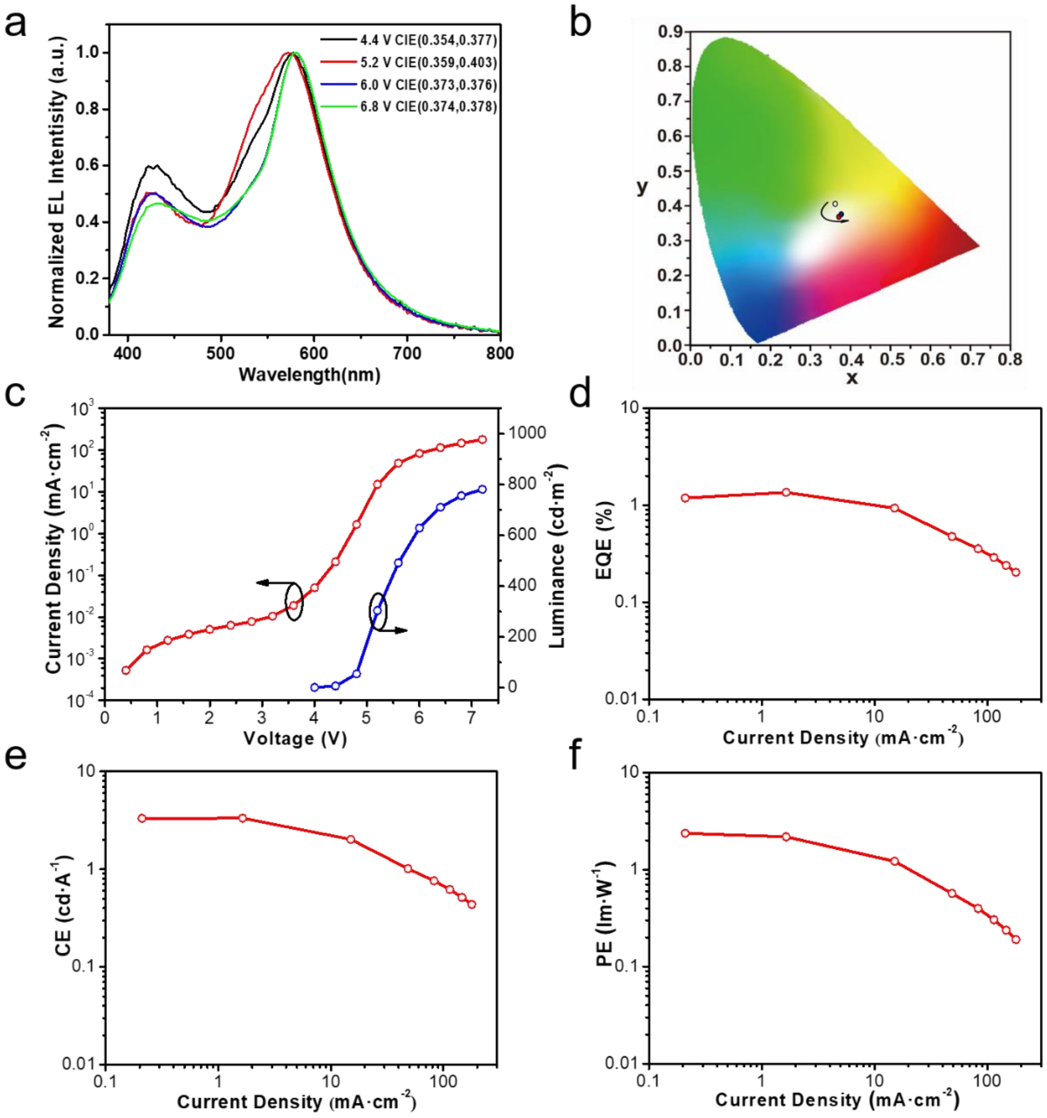

Supplementary Figure 29. Electroluminescence performance of device 3 based on S-**BINAP**: (a) EL spectrum under different operated voltages; (b) The CIE coordinates; (c) current density-voltage-luminance curve; (d) external quantum efficiency- current density curve; (e) current efficiency - current density curve; (f) power efficiency - current density curve.

Supplementary Table 7. The comparison of devices performances between reported RTP-OLEDs and this work.

Compound	Device configuration	λ_{EL} (nm)	EQE (%)	CE_{max} ($cd{\cdot}A^{-1}$)	CIE (x,y)	L_{max} ($cd{\cdot}m^{-2}$)	Ref.
a	ITO / NPB(40 nm) / T SPBA (10 nm) / CBP:10% a (20 nm) / TPBI(50 nm) / LiF (1 nm) / Al (10 nm)	427	0.6	1.0	-	1700	[11]
b	ITO / PEDOT:PSS / PVK:PBD:b /TmPyPB / Liq / Al	510	-	-	-	194	[12]
c	ITO / PEDOT:PSS / TPD/PVK: **c**/PBD / CsF / Al	-	0.0254	-	-	-	[13]
d	ITO / PEDOT:PSS / TPD / PVK:**d** / PBD / CsF / Al	-	0.00558	-	-	-	[13]
e	ITO / NPD (30 nm) / mCP (10 nm) / 1% e:CzSte (30 nm) / TPBi (60 nm) / LiF (0.8 nm) / Al (80 nm)	450	1	-	-	-	[14]
f	ITO / PEDOT:PSS / NPB / f / BCP / Alq_3 / LiF /Al	-	-	-	0.31, 0.44	20	[15]
g	ITO/PEDOT:PSS / NPB / g/BCP /Alq_3 / LiF / Al	-	-	-	0.29, 0.35	110	[15]
h	ITO / PEDOT:PSS / TAPC / mCP / TPBi:27QNX:h / TSPO1 / TPBI / LiF / Al	583	0.11	-	-	22.4	[16]
i	PEDOT:PSS/ TCTA-i /TPBI /Cathod	510	-	-	-	570	[17]
j※	ITO / NPB (25 nm) / TCTA (10 nm) / 10% **j**:TRZ-BIM (20 nm) / TPBI (40 nm) / LiF (1 nm) / Al (100 nm)	570	11.5	33.8	0.46, 0.52	18500	[18]
k	TAPC/CBP:K/BPen	464,569,601	0.2	-	-	-	[19]
l	ITO / MoO_3 (3 nm) / mCP (10 nm) / **1** (100 nm) / TBPi (20 nm) / LiF (0.5 nm) / Al (100 nm)	-	0.6		0.319, 0.270	925	[20]
m	ITO / PEDOT:PSS (35nm) / TAPC (40nm) / mCP (7nm) / rac-BINAP (20nm) / TmPyPB (40nm) / CsF (1.2nm) / Al (120nm)	510,586	1.59	3.18	0.371, 0.441	934	This work
n	ITO / PEDOT:PSS (35nm) / TAPC (40nm) / mCP (7nm) / S-BINAP (20nm) / TmPyPB (40nm) / CsF (1.2nm) / Al (120nm)	425,587	1.36	3.33	0.373, 0.376	781	This work
0	ITO / PEDOT:PSS (35nm) / TAPC (40nm) / mCP (7nm) / S-BINAP (20nm, annealed at 100 ℃ for 5 min) / TmPyPB (40nm) / CsF (1.2nm) / Al(120nm)	482,580	1.58	3.51	0.453, 0.386	1026	This work

-The data didn't appear in the corresponding paper. ※the paper can't offer a precise mechanism for the phenomenon of TRZ-BIM enhanced RTP. Note that the structures of compounds demonstrated in literature are shown in the **Supplementary Figure 30** below.

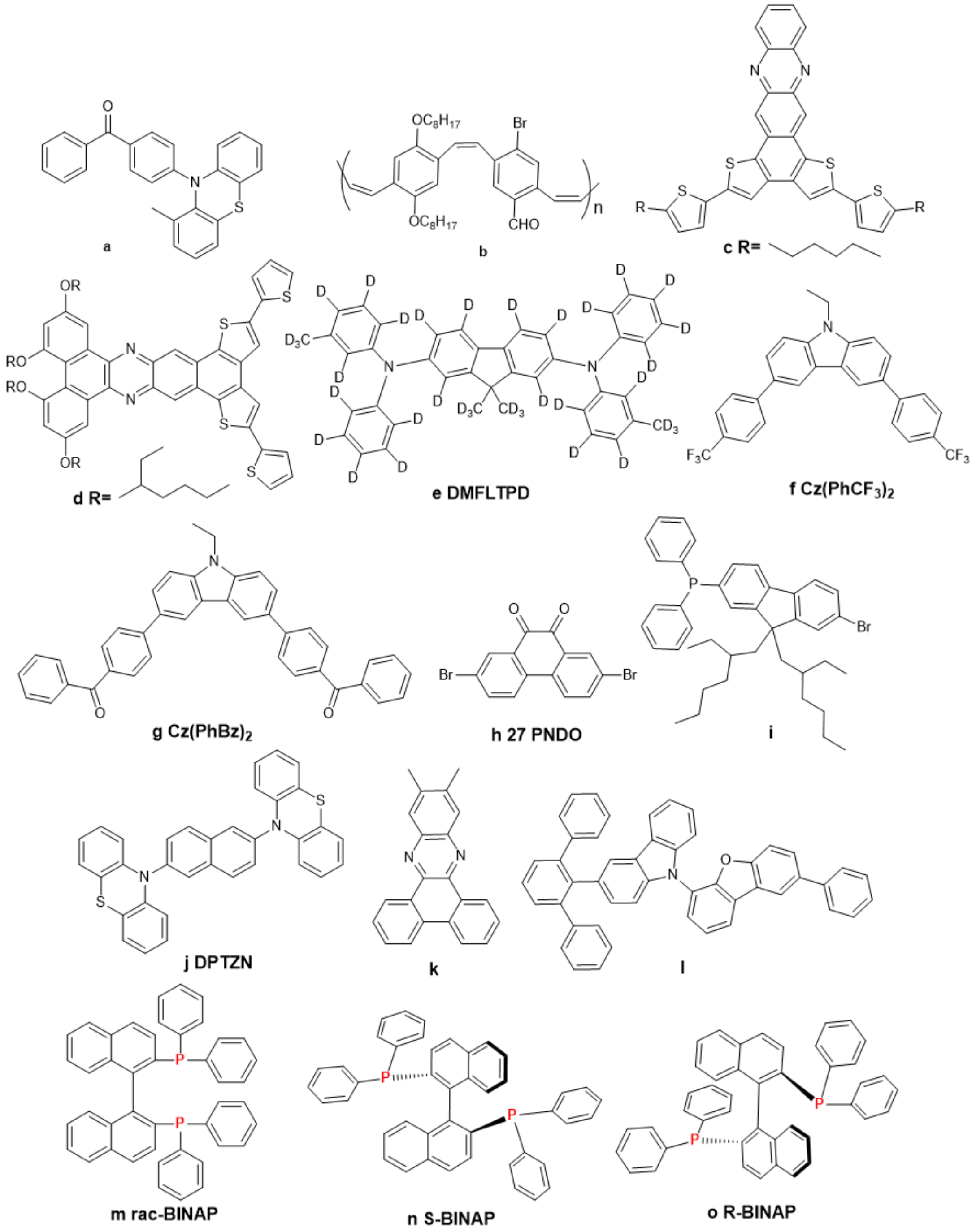

Supplementary Figure 30. The reported molecular structures used in OLEDs. Please refer to **Supplementary Table 7**.

Supplementary References:

1. Wu, X. *et al.* Highly efficient near-infrared emission from binuclear cyclo-metalated platinum complexes bridged with 5-(4-octyloxyphenyl)-1,3,4-oxadiazole-2-thiol in PLEDs. *Org. Electron.* **13**, 932-937 (2012).
2. Dzebo, D., Börjesson, K., Gray, V., Moth-Poulsen, K. & Albinsson, B. Intramolecular triplet–triplet annihilation upconversion in 9,10-diphenylanthracene oligomers and dendrimers. *J. Phys. Chem. C* **120**, 23397-23406 (2016).
3. Chen, X. *et al.* Versatile room-temperature-phosphorescent materials prepared from N-substituted naphthalimides: emission enhancement and chemical conjugation. *Angew. Chem. Int. Ed.* **55**, 9872-9876 (2016).
4. Wu, H. *et al.* Tuning for visible fluorescence and near-infrared phosphorescence on a unimolecular mechanically sensitive platform via adjustable ch−π interaction. *ACS Appl. Mater. Interfaces* **9**, 3865-3872 (2017).
5. Gutierrez, D. G., Sazama, T. G., Wu, T., Baldo, M. A. & Swager, M. T. *J. Org. Chem.* **81**, 4789-4796 (2016).
6. Fateminia, S. M. A. *et al.* Organic nanocrystals with bright red persistent room‐temperature phosphorescence for biological applications. *Angew. Chem. Int. Ed.* **56**, 12160-12164 (2017).
7. Shi, H. *et al.* A Highly efficient red metal-free organic phosphor for time-resolved luminescence imaging and photodynamic therapy. *ACS Appl. Mater. Interfaces* **11**, 18103-18110 (2019).
8. Wang, X.-F. *et al.* Pure organic room temperature phosphorescence from unique micelle‐assisted assembly of nanocrystals in water. *Adv. Funct. Mater.* **30**, 1907282 (2020).
9. Bhattacharjee, I., Acharya, N., Karmakar, S. & Ray, D. Room-temperature orange-red phosphorescence by way of intermolecular charge transfer in single-component phenoxazine–quinoline conjugates and chemical sensing. *J. Phys. Chem. C* **122**, 21589−21597 (2018).
10. Du, J. *et al.* Red room-temperature phosphorescent diimides: molecular design. Preprint at https://chemrxiv.org/articles/Red_Room-Temperature_Phosphorescent_Diimides_Molecular_Design_and_Application/9784865/1 (2019).
11. Chen, C. *et al.* Intramolecular charge transfer controls switching between room temperature phosphorescence and thermally activated delayed fluorescence. *Angew. Chem. Int. Ed.* **130**, 16645-16649 (2018).
12. He, Y., Cheng, N., Xu, X., Fu, J. & Wang, J.-A. A high efficiency pure organic room temperature phosphorescence polymer PPV derivative for OLED. *Org. Electron.* **64**, 247-251 (2019).
13. Ratzke, W. *et al.* Effect of conjugation pathway in metal-free room-temperature dual singlet–triplet emitters for organic light-emitting diodes. *J. Phys. Chem. Lett.* **7**, 4802-4808 (2016).
14. Kabe, R., Notsuka, N., Yoshida, K. & Adachi, C. Afterglow organic light-emitting diode. *Adv. Mater.* **28**, 655-660 (2016).
15. Krishna, A., Darshan, V., Suresh, H. C., Narayanan Unni, N. K. & Varma, L. R. Solution processable carbazole derivatives for dopant free single molecule white electroluminescence by room temperature phosphorescence. *J. Photoch. Photobi. A* **360**,

249-254 (2018).
16. Lee, R. D., Han, H. S. & Lee, Y. J. Metal-free and purely organic phosphorescent light-emitting diodes using phosphorescence harvesting hosts and organic phosphorescent emitters. *J. Mater. Chem. C* **7**, 11500-11506 (2019).
17. Anzenbacher, P., Pérez-Bolívar, C., Takizawa, S. & Brega, V. Room-temperature electrophosphorescence from an all-organic material. *J. Lumin.* **180**, 111-116 (2016).
18. Wang, J. *et al*. Purely organic phosphorescence emitter-based efficient electroluminescence devices. *J. Phys. Chem. Lett.* **10**, 5983-5988 (2019).
19. Ratzke, W., Bange, S. & Lupton, M. J. Direct detection of singlet-triplet interconversion in oled magnetoelectroluminescence with a metal-free fluorescence-phosphorescence dual emitter. *Phys. Rev. Appl.* **9**, 054038 (2018).
20. Tabata, K., Yamada, T., Kita, H. & Yamamoto, Y. Carbazole–dibenzofuran dyads as metal-free single-component white-color photoemitters. *Adv. Funct. Mater.* **29**, 1805824 (2019).
